# Targeting growth hormone receptor in human melanoma cells attenuates tumor progression and epithelial mesenchymal transition via suppression of multiple oncogenic pathways

**DOI:** 10.18632/oncotarget.15375

**Published:** 2017-02-16

**Authors:** Reetobrata Basu, Shiyong Wu, John J. Kopchick

**Affiliations:** ^1^ Edison Biotechnology Institute, Ohio University, Athens, Ohio, USA; ^2^ Molecular and Cell Biology Program, Ohio University, Athens, Ohio, USA; ^3^ Ohio University Heritage College of Osteopathic Medicine, Athens, Ohio, USA

**Keywords:** growth hormone (GH), growth hormone receptor (GHR), melanoma, cancer, IGF-1

## Abstract

Recent reports have confirmed highest levels of growth hormone (GH) receptor (GHR) transcripts in melanoma, one of the most aggressive forms of human cancer. Yet the mechanism of GH action in melanoma remains mostly unknown. Here, using human malignant melanoma cells, we examined the effects of GH excess or siRNA mediated GHR knock-down (GHRKD) on tumor proliferation, migration and invasion. GH promoted melanoma progression while GHRKD attenuated the same. Western blot analysis revealed drastic modulation of multiple oncogenic signaling pathways (JAK2, STAT1, STAT3, STAT5, AKT, mTOR, SRC and ERK1/2) following addition of GH or GHRKD. Further, we show that GH excess upregulates expression of markers of epithelial mesenchymal transition in human melanoma, while the effects were reversed by GHRKD. Interestingly, we observed consistent expression of GH transcript in the melanoma cells as well as marked modulation of the IGF receptors and binding proteins (IGF1R, IGF2R, IR, IGFBP2, IGFBP3) and the oncogenic HGF-MET mRNA, in response to excess GH or GHRKD. Our study thus identifies the mechanistic model of GH-GHR action in human melanoma and validates it as an important pharmacological target of intervention.

## INTRODUCTION

In the current year, melanoma is considered the most aggressive and treatment-resistant form of human skin cancer with an annual incidence of 76,380 in 2016 [1] and a total of approximately 1,000,000 patients in the USA. The numbers of new cases have been rising steadily in the last 30 years, during which the five-year survival rates increased from 86% (1985) to 93% (2012), albeit with a poor quality of life [2]. The estimated mortality from melanoma in the US in 2016 is 10,130 and includes children, adolescents and adults [1]. In spite of the recent breakthroughs in targeted and immunotherapy in melanoma [3, 4], there is a standing need [5–8] for identification of novel targets in all forms of cancer, especially melanoma.

Over the last few decades, the tumor driving properties of GH and GHR had been established in cancers of the breast, colon and prostate [9–12]. The presence of growth hormone receptor (GHR) RNA in human skin cells, especially melanocytes, was reported more than 20 years ago [13], followed by identification of autocrine levels of GH as well as IGF1 in normal and basal cell carcinoma [14]. Along with several reports of elevated GHR RNA and proteins in human melanoma biopsies [15–19], the melanoma cell cycle was also considered to be under an orchestrated regulation of endogenous GH, prolactin (PRL) and adrenocorticotropic hormone (ACTH) [20]. Moreover, primary human melanoma specimens were even found to have high levels of GH releasing hormone (GHRH) receptor (GHRHR) [21], while GHRH-analogs were successful in suppressing malignant melanoma growth *in vivo* [22]. GH action is mediated by binding to a pre-dimerized cognate receptor [GH receptor (GHR)], and may involve direct or indirect activation of well-known intracellular signaling pathways downstream of JAK2 as well as the SRC family kinases [23–34]. These pathways including ERK1/2, STAT1, STAT3, STAT5, AKT and mTOR are known to drive the tumoral progression in melanoma cells [35] and are found to be crucial in the interactions of melanoma with its microenvironment and progression to metastasis [36]. Therefore, it was reasonable to hypothesize that GH putatively occupies a central regulatory role in melanoma cell physiology and the GHR can be targeted to abrogate multiple mechanisms of growth and progression of this type of cancer. Yet no definitive studies have investigated or confirmed the plausible mechanisms and extent of GH action in malignant melanoma or the mediators involved therein.

In this project we assessed the effects of siRNA mediated GHR- knock-down (GHRKD) or of excess GH on four human melanoma cell lines selected from the NCI60 panel of human cancer cells and which were also part of a recent report identifying high levels of GHR in human melanoma cells [19]. Tumoral phenotypes of migration, invasion and proliferation were upregulated by GH excess and downregulated by GHRKD. Our RT-qPCR and western blot analysis revealed that critical oncogenic signaling networks in the melanoma cell are GH-dependent and were significantly suppressed when the GHR was targeted and reduced. This resulted in regressive tumoral phenotypes including a reversal in the expressions of markers of epithelial mesenchymal transition which is a critical event in the initiation of metastatic and chemoresistance properties in cancer [37–40]. Our observations collectively present a mechanistic model of GH-GHR action regulating multiple aspects of melanoma progression.

## RESULTS

### GHRKD suppresses human melanoma cell migration, invasion, colony formation and proliferation

The four human melanoma cells selected for this study have been reported to express GHR and are responsive to exogenous hGH treatment [19]. Prior to investigation of GH effect, we intended to confirm the presence and efficient knock-down of the GHR on these cells. Our RT-qPCR analysis of RNA confirmed high levels of GHR RNA in all four melanoma cells which were reduced by almost 90% following GHR-KD (Supplementary Figure 1a). Western-blot analyses of lysates of GHRKD cells also showed an 75%-90% decrease in GHR protein following the siRNA treatment, when compared to the corresponding scramble(scr)-siRNA transfected controls (Supplementary Figure 1 (b, c)) We further validated our results using immunofluorescence (IF) staining for GHR on these cells, following GHRKD. We observed differential yet high levels of expression of GHR in the cells (Supplementary Figure 1 (d, e, f)), with the GHR protein level increasing in order from SKMEL-5, MDAMB-435, MALME-3M and SKMEL-28 (data not shown). Following transfection with GHR-siRNA, the GHR IF levels reduced markedly, indicating reduced GHR protein expression compared to the scr-siRNA treated controls (Supplementary Figure 1 (d, e, f)).

**Figure 1:**
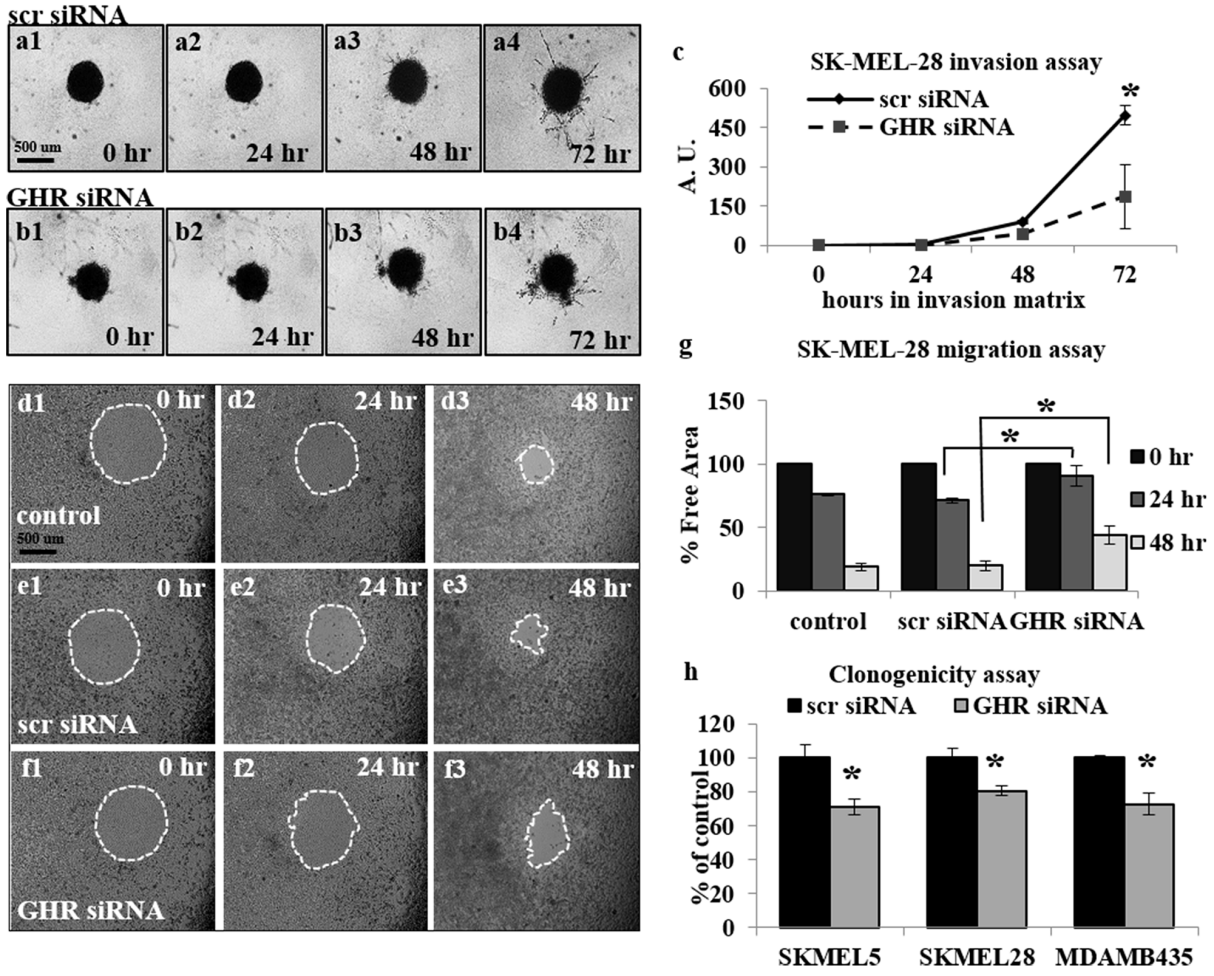
Growth hormone receptor knock-down (GHRKD) attenuates invasion, migration and clonogenicity in human melanoma cells **a-c**. SK-MEL-28 cells transfected with scramble (scr) (a1-a4) or GHR-siRNA (b1-b4) were seeded onto U-bottom 96-well plates at 5000 cells/well and allowed to form a spheroid. A hydrogel invasion matrix was added above the spheroid and cells were monitored for up to 72 hr. in presence of 50 ng/mL hGH. Total pixels representing structural extensions from the spheroid were calculated using ImageJ software and reflected the invasive ability of the melanoma cells (c). A significant decrease in spheroid invasion was noted following GHRKD. **d-g**. SK-MEL-28 cells transfected with scr- (e1-e3) or GHR-siRNA (f1-f3) as well as un-transfected controls (d1-d3) were allowed to migrate into a 0.68 mm circular spot at the center of the well, in presence of 50 ng/mL hGH for up to 48 hr. The percentage free area was calculated using ImageJ software and reflected the decrease/inhibition in migration (g). A significant decrease in migration was noted following GHRKD. Similar results for migration and invasion assays with MALME-3M, MDA-MB-435 and SK-MEL-5 cells are presented in Supplementary Figure 2. **h**. SK-MEL-5, SK-MEL-28 and MDA-MB-435 cells transfected with 20 nM scramble or GHR-siRNA were allowed to form colonies on soft agar for 7 days in presence of 50 ng/mL hGH. The cells were lysed at the end time point and total DNA was quantified using a fluorescent readout. A significant decrease in total number of colonies was noted following GHRKD. [*, p < 0.05, Students t-test, n = 3].

**Figure 2:**
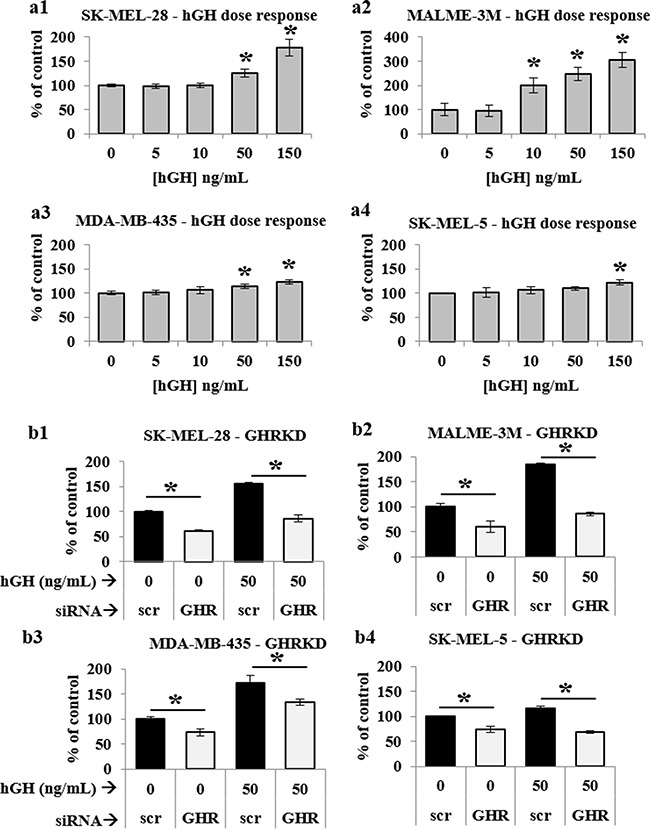
GH-excess promotes and GHRKD attenuates human melanoma cell proliferation **a1**. SK-MEL-28, **a2**. MALME-3M, **a3**. MDA-MB-435 and **a4**. SK-MEL-5 cells were treated with increasing doses of hGH for 48 hr. and cell proliferation was estimated using a resazurin-based metabolic assay. A significant increase in cell proliferation was noted at and above 50 ng/mL hGH treatment. **b1**. SK-MEL-28, **b2**. MALME-3M, **b3**. MDA-MB-435 and **b4**. SK-MEL-5 cells were transfected with 20 nM scramble or GHR-siRNA for 24 hr. and grown for 48 hr. in presence or absence of 50 ng/mL hGH. Cell proliferation was estimated using resazurin-based metabolic assay. A significant decrease in cell proliferation was noted following GHRKD. Averages of at least four independent experiments performed in quadruplicate were taken. [*, p < 0.05, Students t-test].

After confirming successful GHRKD, we analyzed its effect on tumoral phenotypes of proliferation, migration, invasion and clonogenicity. Migration and invasion are critical parameters in tumoral interaction with its microenvironment and cancer metastasis [41, 42]; and different assays are employed to quantify these parameters [43, 44]. In our choice of an appropriate assay, we considered the fact that siRNA mediated knock-down of gene expression is stable up to seven days following transfection. Therefore, to analyze the effects of GHRKD within a relevant time, we chose a commercially available 3-dimensional spheroid assay with a three-day time-point to visualize and quantitate the invasion of melanoma spheroids into a basement membrane protein containing hydrogel matrix with all four cell types, starting 48 hr. post-transfection with scr- or GHR-siRNA. Invasion capacity decreased by a minimum of 28% in MDA-MB-435 cells to as much as 62% in SK-MEL-28 following GHRKD (Figure 1 (a-c), Supplementary Figure 2 (j-l)). To assay the migratory capacity of the melanoma cell lines, the transfected cells could converge on a small circular area in the center of the culture well for up to 48 hr. The percentage free area at the end time point was calculated using ImageJ. We observed a 2-fold reduction in migration level of SK-MEL-28 cells following GHRKD, while for MALME-3M cells the difference was 15-fold when compared against scr-siRNA treated controls (Figures 1 (d-g), Supplementary Figure 2 (a-i)). We also performed the colony formation on soft agar assay which is a perhaps the most widely used method for evaluating the malignant transformation of cells [45] with a high-throughput fluorescent readout. Our experiments showed a significant reduction ranging from 19% (SK-MEL-28) to 28% (SK-MEL-5) in colony formation following GHRKD, despite the presence of hGH in the media (Figure 1 (h)). We did not observe a marked increase in melanoma migration, invasion and clonogenicity on incubation with excess GH (up to 150 ng/mL) although there was a trend towards increase (data not shown). We next evaluated the cell proliferation of the four melanoma cell lines in response to increasing doses (5, 50 and 150 ng/mL) of recombinant hGH. A significant difference in cell proliferation was observed at and above GH concentration of 50 ng/mL. GH-excess induced increase in cell proliferation ranged between 10% (SK-MEL-5) to as much as 248% (MALME-3M) at 50 ng/mL hGH; while at the supra-physiological levels (150 ng/mL), the increase in proliferation ranged from 22% (SK-MEL-5) to more than 300% (MALME-3M) (Figure 2 (a1-a4)). We observed a similar pronounced drop in proliferation levels in all the cell lines when GHR was knocked down. Melanoma cell proliferation decreased by 24% (MDA-MB-435) to 40% (MALME-3M) even when no GH was added externally; while the trend remained similar even when 50 ng/mL hGH was present in the media (Figure 2 (b1-b4)). The results support previous observations and hypotheses [20] and clearly indicate that human melanoma cells utilize GH-GHR interaction to drive aggressive tumor phenotypes. We next investigated possible intracellular signaling networks under GH control that were responsible for translating the GH-GHR interaction to our described effects on tumor progression.

### GH-GHR action regulates phosphorylation states of critical intracellular signaling intermediates in human melanoma cells

**Figure 3:**
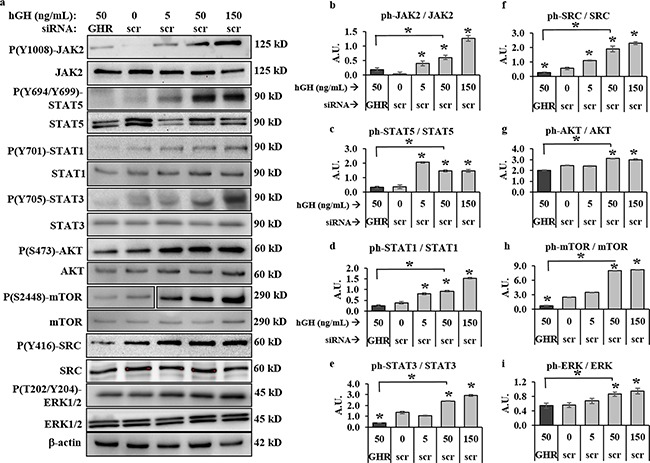
GH-excess promotes and GHRKD attenuates multiple critical intracellular signaling pathways in human melanoma cells **a**. Representative images of western blot (WB) analyses of phosphorylation levels **b**. JAK2, **c**. STAT5, **d**. STAT1, **e**. STAT3, **f**. SRC, **g**. AKT, **h**. mTOR and **i**. ERK1/2, in excess human-GH treated or GHRKD human melanoma cell lysates. SK-MEL-28 cells, 24 hr post-transfection with either scramble (scr)-siRNA or GHR-siRNA were treated for ten mins with GH and lysed as described. WB was performed using appropriate antibodies. Densitometry analyses of individual blots was performed using ImageJ software and the ratio of phosphorylated vs. total protein levels against untreated scr-siRNA transfected controls. Overall, excess GH increased while GHRKD decreased phosphorylation states. Similar results for MALME-3M, MDA-MB-435 and SK-MEL-5 human melanoma cells are presented in Supplementary Figure 3. Blots from individual experiments were quantified and the mean of three blots per antibody was taken. Protein levels were normalized against expression of β-actin. [*, p < 0.05, Students t test, n = 3].

To assess the effects of GHRKD on the activation states of GH regulated shared oncogenic signaling pathways, we treated scr- or GHR-siRNA transfected human melanoma cells, at 60 hr. post-transfection, with 50 ng/mL hGH for 20 minutes and phosphorylation levels of intracellular signaling intermediates were analyzed by western blot (WB). We observed a clear dependence of key signaling pathways on the GH-GHR interaction in all four human melanoma cells (Figure 3a, Supplementary Figure 3i). Densitometry analyses revealed that GH induced a robust increase in phosphorylation levels of JAK2 (Figure 3b, Supplementary Figure 3a) as well as of SRC (Figure 3f, Supplementary Figure 3e) supporting previous findings in other cellular models [26, 31, 32]. In fact, the increases in GH-induced phosphorylation of JAK2 [1.7-fold (SK-MEL-5) to 6.1-fold (SK-MEL-28)] and SRC [1.7 fold (MALME-3M) to 3.5-fold (SK-MEL-28)], were found to be dose-dependent (Figure 3 (b, f)). Even in presence of 50 ng/mL hGH in the medium, GHRKD resulted in as much as 75%-90% lower activation of JAK2 and SRC than corresponding control, often to below the basal activation states observed across the four cell lines (Figure 3 (b, f)).

We additionally found other routes of GH induced signaling downstream of JAK2 and SRC [24–32], including GH-induced increases in phosphorylation states of STAT5 (Figure 3c, Supplementary Figure 3b), STAT1 (Figure 3d, Supplementary Figure 3c), STAT3 (Figure 3e, Supplementary Figure 3d) as well as of AKT (Figure 3g, Supplementary Figure 3f), mTOR (Figure 3h, Supplementary Figure 3g), and ERK1/2 (Figure 3i, Supplementary Figure 3h) in all four human melanoma cell lines. STAT5 phosphorylation increased by 4.1-(SK-MEL-28) to 5.8-fold (MALME-3M) at 50 ng/mL hGH; while GHRKD effected an 80%-90% reduction of the same (Figure 3c, Supplementary Figure 3b). STAT1 and STAT3 phosphorylation levels were markedly upregulated at 50 ng/mL hGH particularly in MALME-3M (2.2-fold and 11.9-fold respectively) and SK-MEL-28 (2.4-fold and 1.9-fold respectively); while GHRKD suppressed phosphorylation significantly across all four cells (Figure 3 (d, e), Supplementary Figure 3 (c, d)). Activated (tyrosine phosphorylated) GHR, in presence of 50 ng/mL hGH, was found to increase AKT and its downstream target mTOR phosphorylation level rates up to 4-fold and 15-fold respectively in SK-MEL-5 cells, while GHRKD suppressed the same by more than 90% in all cases (Figure 3 (f, g)). The ERK1/2 levels were similarly upregulated by GH in three off four melanoma cell lines, with a 5-fold increase in SK-MEL-5 cells (Supplementary Figure 3h). Consistent with our previous observations, GHRKD also suppressed ERK1/2 phosphorylation by 80% in human melanoma cell lines, even in presence of GH. These signaling pathways are well-known oncogenic drivers in several human cancers, especially melanoma [46–54]. Thus, our results at both excess GH and GHR depletion, show that the GH-GHR pair regulates aggressive tumor phenotypes by exerting extensive control over the activation states of important oncogenic signaling mediators.

### GH-GHR action is a driver of EMT in human melanoma cells

**Figure 4:**
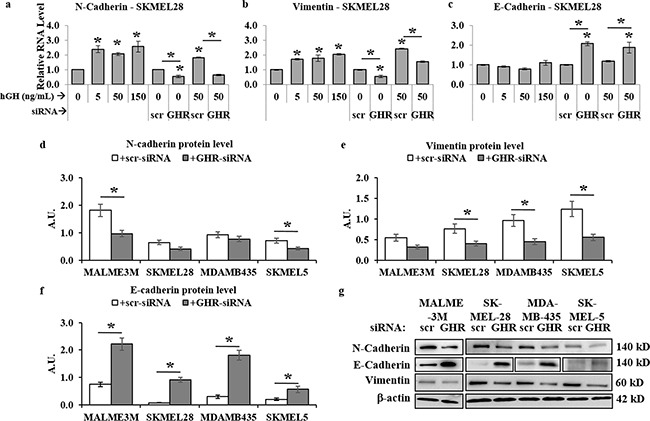
GH-excess promotes and GHRKD attenuates markers of epithelial mesenchymal transition (EMT) in human melanoma cells **a-c**. Relative RNA expression was quantified for N-cadherin (a), vimentin (b) and E-cadherin (c) in SK-MEL-28 melanoma cells following addition of 5, 50 and 150 ng/mL hGH or 24 hr following GHRKD, in presence or absence of 0 and 50 ng/mL hGH. Similar results for MALME-3M, MDA-MB-435 and SK-MEL-5 human melanoma cells are presented in Supplementary Figure 4. In all cases, RNA expressions were normalized against β-actin and GAPDH values as reference genes and compared against untreated control. [*, p < 0.05, Wilcoxon sign rank test, n = 4] **d-f**. Densitometry analyses of relative protein expressions of N-cadherin (d), vimentin (e), and E-cadherin (f) as estimated by western blot (WB) of lysates of SK-MEL-28, MALME-3M, MDA-MB-435 and SK-MEL-5 cells, collected 60 hr post-transfection with either scramble (scr)-siRNA or GHR-siRNA in presence of GH. **g**. Representative images of WB analyses of phosphorylation levels of N-cadherin, vimentin and E-cadherin in four melanoma cell lines. WB was performed using appropriate antibodies. Densitometry analyses of individual blots was performed using ImageJ software and the ratio of phosphorylated vs. total protein levels against untreated scr-siRNA transfected controls. Overall, excess GH promoted while GHRKD reversed EMT in human melanoma cells. Blots from individual experiments were quantified and the mean of three blots per antibody was taken. Protein levels were normalized against expression of β-actin. [*, p < 0.05, Students t test, n = 3].

Recent results reported autocrine GH can mediate direct regulation of EMT via activation of the miRNA-96-182-183 cluster [55]. Our observations described above, as well as previous reports of abundant expression of GHR and readily detectable RNA levels of GH in all four melanoma cells [13–19], in addition to our observation of distinct growth hormone regulation of JAK2, SRC, STAT5, STAT3, AKT, mTOR and ERK1/2 pathways [56–60] – all converge upon initiation and progression of EMT in several forms of cancer. This naturally prompted us to query at changes in known markers of EMT. Our RT-qPCR analysis of 24 hr hGH treated human melanoma cells showed a significant dose-dependent increase of mesenchymal markers N-cadherin [> 2-fold in SK-MEL-28, Figure 4a, Supplementary Figure 4 (a, d, g)]] and vimentin [>2-fold in SK-MEL-28, Figure 4b, Supplementary Figure 4 (b, e, h)] RNA. On the other hand, GHRKD reduced N-cadherin and vimentin RNA levels by as much as 40% (Figure 4 (a, b)). GH significantly suppressed the epithelial marker E-cadherin RNA levels in MDA-MB-435 cells while GHRKD resulted in a 2-fold (SK-MEL-28) to 3-fold (MDA-MB-435) increase (Figure 4c, Supplementary Figure 4f). Western-blot analysis was consistent with our observation of RNA levels. While the effect of GH excess in changing protein levels of N-cadherin, vimentin, and E-cadherin was not significant (data not shown), GHRKD caused marked downregulation (∼50%) of N-cadherin (Figure 4 (d, g)) and vimentin (Figure 4 (e, g)) proteins in all melanoma cell lines. GHRKD effects were more pronounced in upregulating E-cadherin protein levels where we saw a 2.8-fold (SK-MEL-5) to 11.3-fold (SK-MEL-28) increase following transfection (Figure 4 (f, g)). This set of observations thus identifies a critical and unknown role of GH-GHR action in driving the drug-resistance and metastasis inducing EMT pathway in human melanoma.

### Targeting GHR remodels RNA expression of members of the IGF axis and suppresses oncogenic receptors in human melanoma cells

**Figure 5:**
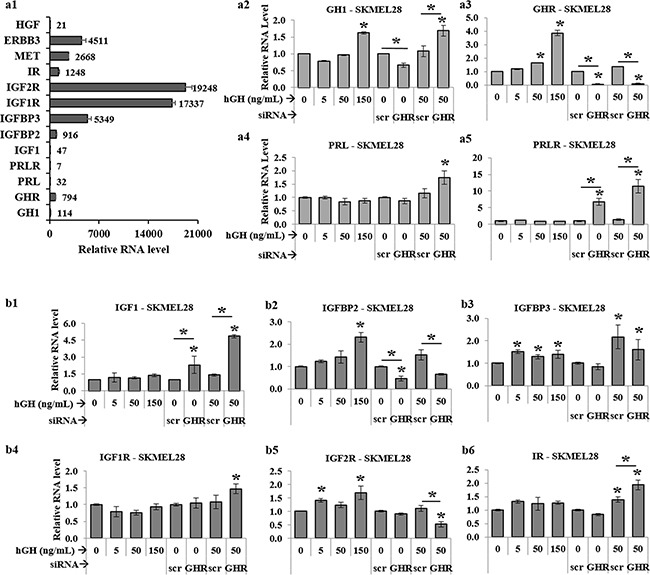
RT-qPCR analysis of GH-IGF axis in human melanoma cells **a1**. Relative levels of RNA expressions for GH, GHR, PRL, PRLR, IGF1, IGFBP2, IGFBP3, IGF1R, IGF2R, IR, MET, ERBB3, and HGF expressed as 1/1000^th^ part of β-actin expression level in SK-MEL-28 melanoma cells. RNA levels of GH **a2**. GHR **a3**. PRL **a4**. and PRLR **a5**. as well as IGF1 **b1**. IGFBP2 **b2**. IGFBP3 **b3**. IGF1R **b4**. IGF2R **b5**. and IR **b6**. following RT-qPCR of RNA extracted from SK-MEL-28 cells following addition of 0, 5, 50 and 150 ng/mL hGH or following GHRKD, in presence or absence of 0 and 50 ng/mL hGH. Results are discussed in the text. Similar results for MALME-3M, MDA-MB-435 and SK-MEL-5 human melanoma cells are presented in Supplementary Figures 5, 6, 7, and 8. In all cases, exogenous hGH treatment was for 24 hr. RNA levels were normalized against expression of β-actin and GAPDH as reference genes. [*, p < 0.05, Wilcoxon sign rank test, n = 4].

GH-GHR induced intracellular signaling is innately associated with that of several other hormones including prolactin (PRL), insulin (Ins), insulin-like growth factors 1 and 2 (IGF-1, and IGF-2) and their respective cognate receptors – PRL receptor (PRLR), insulin receptor (IR), IGF-1 receptor (IGF1R) and IGF-2 receptor (IGF2R) [61, 62]. In addition, GH action is strongly correlated with expression of IGF binding proteins (IGFBP) e.g. IGFBP-2 and IGFBP-3. GH and PRL belong to the same family of class I cytokines, with multiple similar actions on tissues. Additionally, human skin is an extra-pituitary site where both these cytokines and their cognate receptors (GHR and PRLR) are expressed [63]. In view of these facts and our identification of a profound GH mediated regulation of melanoma progression, we wanted to look at presence of endogenous RNA levels of GH as well as PRL and PRLR in human melanoma. In all four melanoma cells, there was a readily detectable expression of GH RNA (Figure 5 (a1, a2), Supplementary Figure 5 (a1, b1, c1)). We could also detect PRL and PRLR but at levels 4-fold and 110-fold lower than GH and GHR levels respectively in SK-MEL-28 cells (Figure 5a1). We observed a similar level of RNA expression across all four melanoma cell lines. In the SK-MEL-28 cells, GHR knockdown resulted in a 1.7-fold rise in GH as well as PRL (Figure 5 (a2, a3)), which was closely reflected in other cell lines (Supplementary Figure 5 (a3, b1, b3, c1)). Intriguingly, we found a 6 to 10-fold rise in PRLR levels with a concomitant 8 to 10-fold drop in GHR levels in SK-MEL-28 cells (Figure 5 (a4, a5)). This significant rise in PRLR with drop in GHR expression was seen in MALME-3M as well as MDA-MB-435 cells (Supplementary Figure 5 (a4, b4)). This data-set hints at a possible compensatory rise in PRL dependency of the melanoma cells, in absence of GH action due to abrogation of GHR expression.

A recent study showed IGF1 to be significantly elevated in circulation of 77 melanoma patients compared to 137 non-melanoma human subjects [64], while IGF1-IGF1R system has been implicated in an autocrine/paracrine regulation of melanoma growth [65]. The network of insulin (Ins), IGF1 and IGF2. their cognate receptors (IR, IGF1R, IGF2R and heterologous pairs) and binding proteins (IGFBPs) are important determinants of melanoma disease progression [66, 67]. In the context of the known importance of each of these molecules in oncogenicity and cancer aggressiveness, we specifically chose to investigate their levels following the perturbation of the GH-GHR axis by either addition of exogenous hGH or GHRKD. We detected no IGF2 or insulin RNA expression in the melanoma cell lines. In agreement with recent comprehensive reports of IGF gene expressions in melanoma [68], we found low levels of IGF1 and very high levels (25-fold greater than GHR) of IGF1R and IGF2R expression (Figure 5a1). Insulin receptors (IRs) were also detected at equivalent levels with GHR (Figure 5a1). Although GH-excess did not cause any consistent variation in the levels of the low amounts of IGF1 RNA detected, GHRKD resulted in a 2-fold [MDA-MB-435] to 8.6-fold [MALME-3M] increase of IGF1 RNA in the four melanoma cells (Figure 5b1, Supplementary Figure 6a, 7a, 8a). Although excess GH caused no consistent variation in their RNA levels, we observed a differential pattern of regulation of the IGF receptors following GHRKD. In SK-MEL-28 (Figure 5b) and MALME-3M (Supplementary Figure 6) cells, GHRKD resulted in a significant increase in the level of IGF1R (1.5-fold) and IR (2-fold) and a significant drop in IGF2R (2-fold). The net effect of this remodeling of IGF receptor distribution might not be a random event but of significance in understanding the dynamicity in targeting receptor tyrosine kinases in melanoma [68]. IGF-binding proteins 2 (IGFBP2) and 3 (IGFBP3) were expressed at relatively high levels in SK-MEL-28 (Figure 5a1) and are known to have differential roles in melanoma progression [69–73]. Importantly, increasing IGFBP2 level has been correlated with progression to metastasis [69] and actively drives proliferation [70] in melanoma. We found an increase in IGFBP2 RNA levels in SK-MEL-28 cells at high GH levels and a significant decrease following GHRKD (Figure 5b2). This 2-fold (SK-MEL-28) to 4-fold (MALME-3M) decrease in IGFBP2 levels after GHRKD, was consistently observed in all melanoma cell lines (Supplementary Figure 6b, S7b, S8b). On the other hand, the IGFBP3 is known to bind IGF1 as well as IGF2 and have an anti-tumor effect in several types of cancers and its concentration decreases markedly in circulation of cancer patients [71]. However, IGFBP3 has also been shown to have an oncogenic potential with drastic increase in expression in cultured human melanoma cells [72]. In our study, except for SK-MEL-28 cells (Figure 5b3), GHRKD increased IGFBP3 levels by as much as 3-fold (Supplementary Figure 6c, 7c, 8c). Thus, our RNA analysis of IGF axis in human melanoma in response to variations in GH action, reflect an intricate pattern of regulation. The overall change in responsiveness to circulating or paracrine insulin and IGFs, induced by blockade of GH action, can be a subject of future studies.

**Figure 6:**
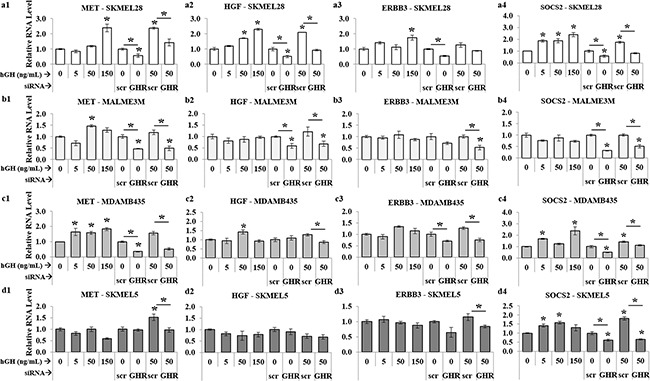
GH-excess increases and GHRKD decreases HGF, MET, ERBB3 RNA-levels in human melanoma cells Relative RNA levels of MET **(a1, b1, c1, d1)**, HGF **(a2, b2, c2, d2)**, and ERBB3 **(a3, b3, c3, d3)**, following RT-qPCR of RNA extracted from SK-MEL-28 (a), MALME-3M (b), MDA-MB-435 (c) and SK-MEL-5 (d) cells following addition of 0, 5, 50 and 150 ng/mL hGH or following GHRKD, in presence or absence of 0 and 50 ng/mL hGH. SOCS2 **(a4, b4, c4, d4)**, was used as an internal control for GH action. Results are discussed in the text. In all cases, exogenous hGH treatment was for 24 hr. RNA levels were normalized against expression of β-actin and GAPDH as reference genes. [*, p < 0.05, Wilcoxon sign rank test, n = 4].

Lastly, in course of studying two spectrums of GH action – GH excess and GHRKD – on human melanoma cells, we also identified significant modulation with response to changes in GH action in a set of three genes - the autocrine system of hepatocyte growth factor (HGF) and its cognate receptor tyrosine kinase MET, and the Erb-B2 receptor tyrosine kinase 3 (ERBB3 or HER3) - reported to be induced by GH in different tissues [74] as well as known to be critical drivers of aggressive disease progression and melanoma drug resistance [75–80]. RT-qPCR analysis of 17 human melanoma samples have identified and subsequent experiments have confirmed the existence of a tumor driving HGF-MET axis [68]. In confirmation of that and other studies, we also found low levels of HGF and consistently high levels of MET and ERBB3 RNA in the four melanoma cell lines used in this study (Figure 5a1). Both HGF and MET expression levels were significantly upregulated in a dose-dependent manner with added GH in SK-MEL-28 (Figure 6a1, a2) and MALME-3M (Figure 6b1, b2) cells to more than 2-fold at maximum GH concentrations. In comparison, this GH-excess mediated increase in expression was more than 50% reduced by GHRKD in all the four cell lines (Figure 6 (a1-d1, a2-d2)). ERBB3 showed a similar upregulation under GH stimulation while GHRKD caused a marked drop in its levels (Figure 6 (a3-d3)). SOCS2 was used as an internal control to monitor GHRKD effects in all RT-qPCR experiments (Figure 6 (a4-d4)). Therefore, our results provide an initial mechanistic detail of GH-GHR activity in melanoma and validates it as a target of interest to abrogate melanoma growth and proliferation.

## DISCUSSION

Human melanoma continues to be a serious cause of global mortality. Excellent new targeted and immune-therapies against human melanoma have been introduced since 2011 [81]. Unfortunately, several reports of resistance against even the latest chemotherapy, [7, 8] point to a pressing need for identifying novel targets in this cancer type which can help in diagnosis and therapy [2]. The highly proliferative effect of an induced autocrine-hGH system in endometrial and mammary carcinoma as well as upregulated migration, anchorage-free growth and propensity to epithelial mesenchymal transition is known [9, 10, 82–84]. The autocrine/paracrine action of human GH in oncogenic incidents has been further established in autocrine GH-driven miR-96-182-183 mediated upregulation of epithelial mesenchymal transition (EMT) in breast cancer [55, 63]. Multiple studies have also reported nuclear localization of GHR, indicating an intracrine role of GH, also considered being particularly relevant in upregulation of tumor proliferation [85, 86]. The studies on the effects of reduced GH-action in humans leading to a significantly reduced cancer burden, have been highlighted by the pioneering studies noting an almost non-existent cancer and diabetes incidence in Laron syndrome patients [87] and in GHD patients[88]. However therapeutic intervention or short life of untreated acromegaly patients hinder us from a similar comparison of cancer incidence in acromegaly patients with increased GH-action [89–92].

In this study, we add to the list of growing evidence implicating GH-GHR pair in human cancer, by presenting mechanistic details of GH-GHR action in human melanoma cells. We observed readily detectable levels of hGH RNA and protein and its cognate GHR on human melanoma cells. Basal level phosphorylation of GH-regulated intracellular signaling networks like JAK2, STATs 1, 3, 5, ERK1/2, SRC, AKT and mTOR in absence of externally added GH suggested the presence of an autocrine ligand-receptor loop existent and critical in these four melanoma cell lines. This observation concurs with the knowledge of aberrant gene expressions in tumors [63, 93] and indicates that GH-GHR pair could be an important marker of metastatic melanoma. Previous reports of histological analysis of human melanoma have confirmed this. Although none of the published reports phenotype the GHR [94–96], we have very recently been able to evaluate GHR expression in B16F10 mouse melanoma cells and found a high expression of GHR in these cells. The results are presently being verified and studied in detail in appropriate syngeneic mouse-models of variable GH-action as well as by using purified recombinant GHR-antagonist (John Kopchick, personal communication). Endocrine as well as paracrine/autocrine GH appears to directly activate critical intracellular signaling pathways and drive aggressive tumor phenotypes and EMT in human melanoma, as we have presented above. Further dissecting the autocrine vs. intracrine roles of this ligand-receptor pair using human melanoma as a model might be of substantial interest. Skin is an extra-pituitary site of GH as well as PRL expression and autocrine effect [63]. PRL and expression of PRLR on tumor tissues have been strongly implicated in breast and prostate cancers for a considerable time via its mitogenic and angiogenic properties [97–99]. We found low but consistent RNA levels of both PRL and PRLR and consistent marked rise in PRLR levels following GHR-KD in SK-MEL-28, MALME-3M and MDA-MB-435 cells. The presence of excess GH potentiated the effect. PRL-PRLR signaling engages intracellular mediators like JAK2, PI3K, ERK1/2 and STAT5 which overlaps with GHR signaling pathway. Also hGH is known to bind and activate PRLR [97]. The siRNA mediated GHRKD could lead to a compensatory non-canonical binding of GH-PRLR and subsequent downstream signaling. We noticed significant basal phosphorylation of the ERK1/2 and AKT/mTOR components in all four melanoma cell lines which can be explained because of a constitutively active RAS, harboring the V600E mutation in these cell lines. However, on GHRKD, we further observed a significant decrease in ERK1/2, AKT, and mTOR in all cases often below the basal levels, irrespective of presence of hGH. This significant downregulation indicates that suppression of an active autocrine GH-GHR interaction contributes significantly to down regulation of the basal phosphorylation states of these signaling pathways. The residual phosphorylation observed following GHRKD, although significantly low, could be induced by GH binding and activation of PRLR as well as other shared signaling pathways. Importantly, we show that exogenous GH and GHRKD had significant enhancing and suppressing effects respectively, on relevant intracellular signaling pathways. Similarly, the effect of increased PRLR from endocrine or paracrine PRL cannot be ruled out. Like our studies, PRLR-inhibited/down regulated models can provide a mechanistic detail of PRL action and a putative salvage pathway involving GH-PRLR interaction specifically in melanoma. However, we observed that the RNA levels of PRLR in these melanoma cells are more than 100-fold lower than the GHR RNA levels, and we observed no significant variation due to altered autocrine PRL-PRLR level on GHRKD induced effects in either the phosphorylation levels of intracellular signaling intermediates or in the tumoral phenotypes of migration, invasion and proliferation.

The role of IGF axis in human melanoma is also of considerable importance and prompted us to analyze RNA levels of insulin-IGF axis in human melanoma cells during GH-excess and GHRKD. We did not detect any endogenous insulin or IGF2 RNA or protein levels in any of the four melanoma cells tested, but our RT-qPCR studies revealed presence of IGF1 RNA that is supported by recent reports [68]. IGF1 & IGF2 and their cognate receptors are important regulators of multiple human cancers, including melanoma [67, 100–103]. We found high levels of RNA of the corresponding cognate receptors – IR, IGF1R, and IGF2R in all the four melanoma cells studied here. There are yet no known studies to characterize the role of IGF2 action in melanoma but some isolated studies have pointed at a tumor suppressing role of IGF2 in mammary carcinoma cells expressing IGF2R [104]. In our analysis, we found significant suppression of IGF2R on all melanoma cells following GHRKD but a significant rise in IGF1R and IR RNA levels following GHRKD especially when treated with excess GH. A state of insulin resistance, which includes a high level of circulating insulin, has been associated with a higher risk of melanoma incidence [66]. As we report here, the melanoma cells indeed appear to be in a state of heightened insulin/IGF sensitivity via abundant expression of IR, IGF1R and IGF2R as seen in all four melanoma cells in this study. Interestingly, we observed increased levels of IGFBP3 following GHRKD for the four cell lines with a concomitant increase in IGF1 levels. We speculate that this could possibly be an IGF1 mediated increase in IGFBP3 levels, as have been reported in retinal epithelial cells [73]. However, the regulatory role of IGF axis in human melanoma appears to be limited at the early stage of disease progression and not in case of metastatic malignant melanoma [67, 100, 105, 106]. Additionally, as with PRL and PRLR, the base RNA levels of IGF1 were 3-fold lower than GH levels and the modulated-GH-action induced changes in IGF axis apparently had no observable variation on intracellular signaling or tumor phenotypes as described above. However, the role of endocrine or paracrine IGF1, insulin or IGF2 in an entire organism might reveal additional effects and provide for a holistic analysis of this aspect of IGF regulation in melanoma. Overall, in most cancer treatments, achieving a therapeutic reduction in endocrine IGF1 levels appears to be immensely favored in halting tumor progression [107–109]. Moreover, starvation induced reduction in circulating IGF1 is known to preferentially protect normal cells but sensitize melanoma cells to chemotherapy [110]. Our study, along with these facts, indicates an excellent opportunity of using GHR-antagonists in melanoma therapy, which on one hand can potentially reduce tumor cell proliferation by targeting the increased GHR expression on tumor cells and also reduce circulating IGF1 levels by decreasing hepatic IGF1 output [111]. Therefore, our study hints at the mechanistic rationale of combining GHR antagonism with IGF1R inhibition [112], as a logical treatment combination in malignant human melanoma.

Another important finding of our study was the identification of GH regulation of the autocrine hepatocyte growth factor (HGF) and its cognate receptor MET (or c-MET) on the four melanoma cell lines supporting and adding to recent observation [68]. Although intrinsic RNA levels of HGF were low, there was significant increase when treated with excess hGH in SK-MEL-28 and MDA-MB-435 cells as well as a significant downregulation following GHRKD. Moreover, there was very high RNA levels of the HGF-receptor MET on all four melanoma cells and exhibited a dose dependent rise with added hGH. On the other hand, GHRKD significantly suppressed the same, even in presence of excess hGH. This set of results suggest a possible transcriptional control of MET and HGF expression by hGH. Additionally, the ERBB family members EGFR, ERBB1, ERBB2 and ERBB3 are known to be involved in driving several oncogenic processes in melanoma [36, 77, 113]. We showed here that RNA levels of ERBB3 were upregulated in response to excess GH in SK-MEL-28 and MDA-MB-435 and a consistent suppression occurred following GHRKD. Both MET and EGFR are known to strongly activate the SRC signaling pathway [75, 78]. GH is known to activate EGFR in liver regeneration [114]. Thus, our results indicate a regulatory role of GH on expression of HGF, MET and ERBB3 in human melanoma cells. Identifying the underlying mechanisms of transcriptional regulation and downstream intracellular targets can add value to the extent of dependence of malignant metastatic melanoma on the GH-GHR axis.

STAT3 activation in melanoma drives multiple critical transformations including EMT, angiogenesis and inhibition of apoptosis by increasing expressions of intrinsic oncogenic factors like microphthalmia associated transcription factor (MITF) and also cooperatively induces downstream factors like c-fos [36, 115–117]. Robust GH-mediated STAT3 regulation is of further importance especially in melanoma following the recent report of the implication of the transcription factor in reprogramming senescent melanomal precursors towards tumorigenicity [118] and demands new studies investigating role of GH in cellular reprogramming and cancer initiating cells. STAT3 is also a converging point in signaling networks for multiple different upstream regulators, e.g. SRC, JAK2 as well as ERBB family members like EGF4 and ERBB3 [50]. Our results agree with previous observations of the presence of constitutive activation of SRC and STAT1, 3, and 5 proteins in melanoma tumors. We found the known pattern of GH-induced activation of STAT proteins [119] to be active in melanoma. In our results, the significant decrease of STAT activation below basal levels, even in presence of excess GH, with GHRKD suggests (i) attenuation of the autocrine GH-mediated activation, as well as (ii) sensitivity and dependence of the melanoma cells on GH-GHR interaction and activation of either JAK2 or SRC or both. The presence of basal phosphorylation of both JAK2 and SRC kinases as well as their respective changes with GHRKD and/or excess GH as observed in all cell lines, indicates that both signaling mediators to be highly responsive to GH in melanoma. A separate analysis of the mutually independent roles of JAK2 and SRC in downstream signaling activation and cell fate, similar to studies done in pre-adipocytes and human hepatoma cells [32, 120], would be of interest and value to resolve in further studies. The distinct upregulation of the basal STAT1 phosphorylation levels, agrees with and puts forth GH action to explain observations in recurrent melanoma phenotypes [121]. The STAT5 dependence on GH-GHR induced activation as observed in this study also establishes the role of GH-GHR action in activating STAT5, which is already an established oncogenic driver in melanoma and protects the cell against interferon-based immunotherapies [122, 123]. In melanoma cells, STAT5 acts to mediate resistance to apoptosis and is reported to be activated by both JAK2 and SRC kinases [47]. Thus, our results indicating significant basal activation of JAK2, SRC, STATs 1, 3, and 5, in melanoma aids in resolving finer aspects of STAT1 vs. STAT3 as well as acts as a springboard for dissecting studies on cytokine-induced STAT mediated cross-talks between JAK and SRC pathways [31, 124]. We show that melanoma cells orchestrate increased proliferation, invasion and migration using GH regulated intracellular signaling pathways and also upregulate oncogenic pathways like HGF-MET and ERBB3. However, another and one of the most important role of the GH-GHR axis in melanoma might be in driving the EMT process, as presented above.

EMT plays a physiological role in wound-healing, fibrosis as well as in progression of cancer [36]. Melanomas break free from the homeostatic control of keratinocytes by loss in expression of E-cadherin, upregulation of expression of fibroblast interacting cadherins like N-cadherin, and upregulation of mesenchymal markers like vimentin [36, 125]. Numerous studies have reviewed the importance of EMT in cancer metastasis [37, 126]. Recent research using EMG lineage tracing studies established a critical role of EMT as a regulator of drug resistance in lung cancer [40]. Recent results also reported an autocrine GH mediated direct regulation of EMT via activation of the miRNA-96-182-183 cluster [55]. This provided a sound scientific rationale to investigate the effects of GHRKD on the EMT markers in melanoma cells. Our observations of reappearance or increase of epithelial markers (E-cadherin) and a marked concomitant downregulation of mesenchymal markers like N-cadherin and vimentin following GHRKD, at both RNA and protein levels thus describe a unique role of GH as an important regulator of EMT and aggressive phenotypes of melanoma multi-drug resistance and metastasis. *In vivo* validation of the entire set of our observations in appropriate mouse models is vital in affirming the versatile role of GH in regulating melanoma.

**Figure 7:**
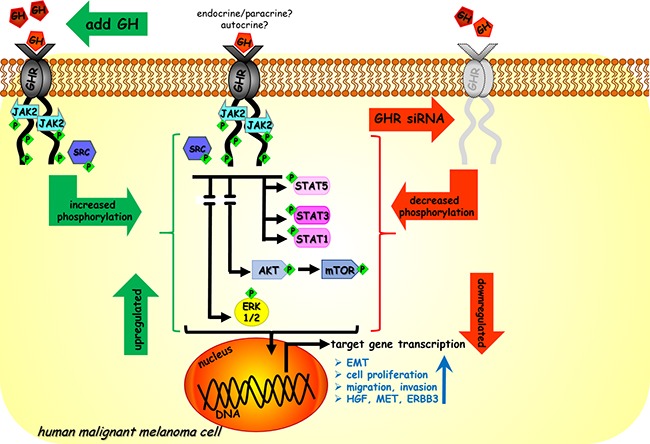
Model of GH regulation in melanoma Endocrine/paracrine/autocrine GH binds to GHR expressed at high levels in human malignant melanoma cells and activates JAK2 and SRC kinases. This leads to downstream activation of STAT1, STAT3, STAT5, ERK1/2, AKT and mTOR. Subsequent transcription of target genes lead to aggressive phenotypes of tumor cell migration, invasion and proliferation and upregulate autocrine HGF-MET loop, ERBB3 and also drives epithelial-mesenchymal-transition. In our study excess GH upregulated (in green) while siRNA mediated GHR knock-down (GHRKD) downregulated (in red) these effects.

In summary, we present a mechanistic model of GH regulation in human malignant melanoma cells (Figure 7). Endocrine or paracrine as well as autocrine GH binds to abundantly expressed GHR on human melanoma and activates the JAK2 as well as SRC kinases. This activation leads to phosphorylation of STAT1, STAT3, STAT5, ERK1/2, AKT and mTOR and goes on to drive EMT and promote invasion, migration, and proliferation for tumor progression. Together our results identify novel regulatory roles of GH in one of the most aggressive and disease-resistant forms of cancer. Using GHRKD, we demonstrate that targeting GHR can be a validated point of intervention in melanoma therapeutics and deserves prompt attention in the present context of continual occurrence of chemotherapy resistance.

## MATERIALS AND METHODS

### Cell culture and GH treatment

Human malignant melanoma cell lines (part of NCI-60 panel of human cancer cells) - SK-MEL-5, SK-MEL-28, MALME-3M, MDA-MB-435S and normal human skin fibroblast cells MALME-3 cells were obtained from American Type Culture Collection (ATCC; Manassas, VA). SK-MEL-5 and SK-MEL-28 were grown and maintained in EMEM media (ATCC), while MALME-3M and MDA-MB-435S were grown in IMDM (ATCC) and RPMI-1640 (ATCC) respectively, as indicated by ATCC protocols. Complete growth media was supplemented with 10% fetal bovine serum (FBS; ATCC) and 1X antibiotic-antimycotic (Thermo Fisher Scientific, Waltham, MA). MALME-3 cells were grown in McCoy's medium (ATCC) supplemented with 15% FBS and 1X antibiotic-antimycotic. Cells were grown at 37C / 5% CO_2_ in a humidified incubator. Half the media was replaced every 48 hr. No hGH was present in the media or added externally unless specifically mentioned. For hGH treatment, 16 hr. after seeding (or 24 hr. post-transfection), the cells were serum-starved for 2 hr. in serum free growth media and hGH (PBS as control) was added at the mentioned concentrations (5, 50, 150 ng/mL). Cells were subsequently incubated for 24 hr. before RNA extraction. Recombinant hGH was purchased from Antibodies Online (Atlanta, GA).

### Transfection

Transfection was performed using siLentFect lipid reagent (Biorad, Hercules, CA) following the manufacturers protocol. Pre-designed siRNA duplexes against human GHR (Origene, Rockville, MD) at different concentrations were evaluated and 20 nM was found to be optimum for decreasing the GHR RNA by >85%. Mock transfections were performed using universal scrambled negative control siRNA-duplex (Origene). TYE-563-fluorescent labeled siRNA duplex (Origene) was used as the transfection control. Cells were trypsinized, counted using a Countess automated cell counter (Life Technologies, Carlsbad, CA) and seeded at 25,000 cells/cm^2^ and allowed to attach overnight, followed by replacing the media with fresh antibiotic free complete growth medium just prior to transfection. A pre-incubated mix of 20nM siRNA duplex (scramble or GHR specific) and siLentFect reagent at 1:1 molar ratio was added to the cells and incubated at 37C / 5% CO_2_. Media was changed to complete growth medium plus antibiotics after 24 hr. RNA was extracted 48 hr post transfection while protein was extracted 60 hr post transfection.

### RNA extraction and RT-qPCR

RNA extraction was done using the IBI-Trizol based total RNA purification kit (MidSci, St. Louis, MO), and reverse transcription was performed using Maxima First Strand cDNA synthesis kit (Thermo Fisher Scientific) following the manufacturers’ protocol. Real time-quantitative PCR and melt curve analysis were performed using Maxima SYBR-Green qPCR master mix (Thermo Fisher Scientific) and a T100 thermal cycler (Biorad). RNA and DNA concentrations were estimated using Nanodrop2000 (Thermo Fisher Scientific) spectrophotometer. Primers were obtained from Sigma-Aldrich (St. Louis, MO) for the following human genes and primer efficiency was confirmed: GAPDH, ACTB β-Actin, GH1, GHR, GHRHR, SOCS2, IGF1, IGF1R, IGF2, IGF2R, PRL, PRLR, Ins, IR, IGFBP2, IGFBP3, EGFR, HGF, MET and ERBB3. Each sample represents a pool of two replicates per experiment: Experiments were done at least three times. Each qPCR for individual genes and every treatment for every cell type was performed in triplicates.

### Protein extraction

Total protein was collected 60 hr post transfection. The conditioned growth media for each treatment type were collected separately for subsequent analysis of secreted proteins. Total protein was extracted from the cells using RIPA buffer (Sigma-Aldrich), mixed with 1.5X Halt protease and phosphatase inhibitor cocktail (Thermo Fisher Scientific), following the manufacturer's protocol. Briefly, cells were washed twice with chilled sterile 1X phosphate buffered saline (PBS). Thereafter, chilled RIPA buffer at 1 mL per million cells were added and incubated for 5 min / 4C. Then the cells were rapidly scraped for cell lysis. The cell lysate was clarified by centrifuging at 8,000Xg / 10 min / 4C and the supernatant was collected and stored at -80C for subsequent use. Each sample was a pool of three replicates per experiment and each experiment was done three times.

Protein concentration was estimated using the Bradford reagent (Sigma-Aldrich) and 1 mg/mL bovine serum albumin (BSA) as standard. Absorbance at 595 nm was measured using Spectramax250 (Molecular Devices, Sunnyvale, CA) and SoftmaxPro v4.7.1 software.

### Western blotting (WB)

Western blotting was performed as described [127]. Briefly cell lysates were separated by SDS-PAGE and transferred to PVDF membranes, blocked with 5% non-fat dry milk or 5% BSA in 1X TBS-T (Tris buffered saline, pH7.2 with 0.1% Triton-X100), incubated with primary antibody (at specific dilutions given below) overnight and finally incubated with corresponding secondary antibodies (at specific dilutions given below) for 2 hr / 25C. Membranes were then washed and treated with West Femto Chemiluminiscence detection reagents (Thermo Fisher Scientific) and the chemiluminiscence signal was captured using a GelDoc (Biorad) fluorescence reader. Densitometry analysis of the blots was done by measured band-intensity from the area-under-curve using ImageJ software.

Primary antibodies at specific dilutions were used to detect the following human proteins: GH (Rabbit, 1:100, Abcam #ab155276), GHR (Mouse, 1:300, SCBT #137185; Goat, 1:100, R&D Systems #AF1210; Rabbit, 1:200, Abcam #ab134078), STAT5 (Rabbit, 1:100, CST #9358S), P(Y694/Y699)-STAT5 (Rabbit, 1:100, ActiveMotif #39617, 39618), P(Y701)-STAT1 (Rabbit, 1:100, CST #7649), P(Y705)-STAT3 (Rabbit, 1:100, CST #9145), STAT3 (Rabbit, 1:200, CST #4904), STAT1 (Rabbit, 1:200, CST #9175), p44/42 MAPK (Erk1/2) (Rabbit, 1:2000, CST #9102S), P-p44/42 MAPK (Erk1/2) (Rabbit, 1:3000, CST #4370P), Akt (Rabbit, 1:2000, CST #4685S), P-Akt (Rabbit, 1:1000, CST #4058S), P-Jak2 (Rabbit, 1:200, GeneTex#61122; Rabbit, 1:100, CST #8082), JAK2 (Mouse, 1:200, Sigma Aldrich # SAB4200483), mTOR (Rabbit, 1:1000, CST #2983), P-mTOR (Ser2448) (Rabbit, 1:2000, CST #5536), P-mTOR (Ser2481) (Rabbit, 1:2000, CST #2974), Raptor (Rabbit, 1:500, CST #2280), Rictor (Rabbit, 1:500, CST#2114), GbL (Rabbit, 1:1000, CST #3274), β-Actin (Goat, 1:3000, SCBT #sc1616), GAPDH (Goat, 1:3000, SCBT #sc20357), P(S1524)-BRCA1 (Rabbit, 1:500, CST#9009), P(S139)-histone H2A.X (Rabbit, 1:1000, CST #9718), histone H2A.X (Rabbit, 1:1000, CST #2595), Caspase-3(Rabbit, 1:1000, CST#9665), cleaved (Asp175)-Caspase-3 (Rabbit, 1:1000, CST #9664), P(Y416)-SFK (Rabbit, 1:200, CST #2101), P(Y416)-SRC (Rabbit, 1:200, CST #6943), SRC (Rabbit, 1:500, AbcaM #47405). Secondary antibodies used were anti-rabbit HRP-linked IgG (Donkey, 1:2000, CST #7074P2; 1:2000, GE #NA934), anti-goat HRP-linked IgG (Donkey, 1:1000, SCBT #sc2020), anti-mouse HRP-linked IgG (Rat, 1:1000, Antibodies Online #ABIN1589975).

### Immunofluorescence (IF)

Cells were seeded at 10,000 cells/cm^2^ in 8-well chamber slides and transfection was performed as described above. Transfection media was replaced with antibiotic containing complete growth media after 24 hr and cells were fixed after 36 hr more (a total of 60 hr post-transfection), and cells were fixed with 4% freshly-prepared formaldehyde (pH6.9) / 15 min / 25C (using 100% methanol for fixation gave equally good results). Cells were permeabilized with 0.2% Triton-X100 in 1X PBS / 15 min / 25C, followed by blocking with 1% BSA / 4 hr / 25C. Incubation time was 12hr / 4C for primary antibody and 2 hr / 25C for secondary antibody. Finally, the slides were washed four times with 1X PBS and the sample was mounted with Fluoroshield mounting medium containing DAPI (Abcam #ab104139, Cambridge, UK), covered with a 60 mm coverslip and the edges were sealed with nail-polish and stored at 4C for microscopy. Microscopic imaging was done using a Nikon Eclipse E600 compound fluorescent microscope fitted with a Nikon DS-Fi1CC camera (Nikon, Tokyo, Japan) and NIS-Elements BR3.2 imaging software. Sera used were rabbit anti-human-Ki67 monoclonal antibody with AlexaFluor488 tag (Abcam #ab154201, 1:300 dilutions); rabbit anti-human GHR monoclonal antibody (Abcam #ab134078, 1:250 dilution); goat anti-rabbit secondary antibody with AlexaFluor488 tag (Life Technologies #R37116, 1:500 dilution).

### Cell proliferation assay

A 1% (w/v) resazurin (Sigma-Aldrich) solution in 1X PBS was made and filter-sterilized. The final concentration of resazurin in the assay was 0.004%. Inside the proliferating cells mildly fluorescent blue resazurin is reduced to a bright pink fluorescent product called resorufin (stable for 4 hr), which is a quantitative measure of the percentage of proliferating cells [128]. In all cases, cells were incubated at 37C / 5% CO_2_ for 45-60 minutes for adequate sensitivity of detection. Briefly, cells were seeded at 10,000 cells/cm^2^ into 96-well plates and transfected as described above. The resazurin assay was performed 60 hr after transfection (unless specified otherwise) and resorufin absorbance was measured at 570 nm (reference wavelength = 600 nm) using Spectramax250 (Molecular Devices) and SoftmaxPro v4.7.1 software.

### Cell migration assay

Cell migration assays are standard methods of estimating the repair and regenerative properties of cells [129]. For our purpose, we used the Radius Cell Migration Assay design from Cell Biolabs (Cell Biolabs #CBA-125, San Diego, CA) and experiments were performed as per manufacturer's protocol. In this assay, a 24-well plate containing a non-toxic, 0.68 mm biocompatible hydrogel spot is present at the center of the well, where cells cannot attach. siRNA treated cells were trypsinized 48 hr. after transfection, counted and seeded at 5000 cells/well in a pretreated hydrogel spot containing 24-well plate. The hydrogel spot was gently removed after 24 hr incubation at 37C / 5% CO_2_. The cells were allowed to migrate for up to 48 hr at 37C / 5% CO_2_. Images were captured every 24 hr using a 4X objective (total magnification 40X) employing an inverted Olympus IX70 microscope fitted with a Retiga 1300 camera (QImaging, Surrey, BC). Total uncovered area at the beginning and end of assay were quantitated using ImageJ software. Experiments were done in triplicates.

### Cell invasion assay

The 96-well 3D spheroid BME cell invasion assay (Trevigen, Gaithersburg, MD) was used to evaluate the ability of cells to invade surrounding tissue. Tumor spheroids are better representatives of tumors *in-vivo*, compared to tumor cells in a Boyden chamber, as is used in multiple invasion assay designs [44]. Briefly, siRNA (scramble or GHR specific) treated melanoma cells were trypsinized 48 hr. after transfection, counted and seeded at 5000 cells/well in a 96-well spheroid formation plate and incubated for 72 hours at 37C / 5% CO_2_ to allow spheroid formation. Thereafter, the invasion matrix was added, followed by 50 ng/mL hGH-containing culture medium as a chemoattractant. The invasive behavior of the cells was monitored every 24 hr. for up to 72 hr. Images were taken every 24 hr using a 4X objective (total magnification 40X) using an inverted Olympus IX70 microscope fitted with a Retiga 1300 camera (QImaging, Surrey, BC). Total pixels at the beginning and end of assay were quantitated using ImageJ software. Experiments were done in triplicate.

### Clonogenicity assay

Colony formation on soft agar or anchorage independent colonization is considered to be a very stringent test for malignant transformation of cells and a hallmark of cancer. Ability of the tumor cell to develop colonies on soft agar reflects a reduced dependence for extracellular growth promoting factors, independence from the control of neighboring cells (like keratinocytes in the case of melanocytes) and infinite capacity to proliferate. For our purpose, we chose the CytoSelect 96-well format (Cell Biolabs #CBA-130, San Diego, CA), which provides a timely and quantitative (fluorimetric) readout of the total colonies formed. Experiments were performed as per the manufacturer's protocol. Briefly, a 0.6% base agar medium containing 1X RPMI-1640 (10% FBS) was prepared and allowed to settle for 30 min / 4C. siRNA treated cells were trypsinized 48 hr. after transfection, counted and seeded at 5000 cells/well in a 0.4% top agar layer also containing 1X RPMI-1640 (10% FBS) and allowed to settle for 15 min / 4C. Finally, 100 uL of pre-warmed culture media containing 50 ug/mL hGH was added on top and incubated for 14 days at 37C / 5% CO_2_. The media was then removed, the agar was solubilized and the cells were lysed in situ. Total DNA content was measured using the CyQuant GR dye (kit component) and fluorescence was measured at 485 (ex) / 520 nm (em) using a spectramax M2 fluorescence plate reader (Molecular Devices) and SoftMax Pro v6.2.1 software. Experiments were done three times and in quadruplicate.

### Statistical analyses

Parametric and non-parametric statistical analyses for comparing RNA levels were done using R software (ver3.0.2). For RT-qPCR analysis of RNA, the levels were first normalized against two reference genes (GAPDH and beta-actin) and the 2^(-ddCt) values were compared by Wilcoxon signed rank test for significance. A p-value less than 0.05 was considered as significant. The densitometry analyses, clonogenicity, migration and invasion, and resazurin based assays, were compared by a paired students T-test and ANOVA was performed (using GraphPad Prism software) to compare for significance (p<0.05 is considered significant).

## SUPPLEMENTARY MATERIALS FIGURES



## References

[1] Melanoma of the Skin - SEER Stat Fact Sheets. http://seer.cancer.gov/statfacts/html/melan.html.

[2] Shain AH, Bastian BC (2016). From melanocytes to melanomas. Nat Rev Cancer.

[3] Sharma A, Shah SR, Illum H, Dowell J (2012). Vemurafenib: targeted inhibition of mutated BRAF for treatment of advanced melanoma and its potential in other malignancies. Drugs.

[4] Luke JJ, Ott PA (2015). PD-1 pathway inhibitors: The next generation of immunotherapy for advanced melanoma. Oncotarget.

[5] Hernandez-Davies JE, Tran TQ, Reid MA, Rosales KR, Lowman XH, Pan M, Moriceau G, Yang Y, Wu J, Lo RS, Kong M (2015). Vemurafenib resistance reprograms melanoma cells towards glutamine dependence. J Transl Med.

[6] Thang ND, Nghia PT, Kumasaka MY, Yajima I, Kato M (2015). Treatment of vemurafenib-resistant SKMEL-28 melanoma cells with paclitaxel. Asian Pac J Cancer Prev.

[7] Bu X, Mahoney KM, Freeman GJ (2016). Learning from PD-1 Resistance: New Combination Strategies. Trends Mol Med.

[8] Zaretsky JM, Garcia-Diaz A, Shin DS, Escuin-Ordinas H, Hugo W, Hu-Lieskovan S, Torrejon DY, Abril-Rodriguez G, Sandoval S, Barthly L, Saco J, Homet Moreno B, Mezzadra R (2016). Mutations Associated with Acquired Resistance to PD-1 Blockade in Melanoma. N Engl J Med.

[9] Perry JK, Emerald BS, Mertani HC, Lobie PE (2006). The oncogenic potential of growth hormone. Growth Horm IGF Res.

[10] Brunet-Dunand SE, Vouyovitch C, Araneda S, Pandey V, Vidal LJ, Print C, Mertani HC, Lobie PE, Perry JK (2009). Autocrine Human Growth Hormone Promotes Tumor Angiogenesis in Mammary Carcinoma. Endocrinology.

[11] Pandey V, Perry JK, Mohankumar KM, Kong XJ, Liu SM, Wu ZS, Mitchell MD, Zhu T, Lobie PE (2008). Autocrine human growth hormone stimulates oncogenicity of endometrial carcinoma cells. Endocrinology.

[12] Kopchick JJ, List EO, Kelder B, Gosney ES, Berryman DE (2014). Evaluation of growth hormone (GH) action in mice: Discovery of GH receptor antagonists and clinical indications. Molecular and Cellular Endocrinology.

[13] Tavakkol A, Elder JT, Griffiths CEM, Cooper KD, Talwar H, Fisher GJ, Keane KM, Foltin SK, Voorhees JJ (1992). Expression of Growth Hormone Receptor, Insulin-Like Growth Factor 1 (IGF-1) and IGF-1 Receptor mRNA and Proteins in Human Skin. The Journal for Investigative Dermatology.

[14] Slominski A, Malarkey WB, Wortsman J, Asa SL, Carlson A (2000). Human skin expresses growth hormone but not the prolactin gene. J Lab Clin Med.

[15] Lincoln DT, Sinowatz F, Kolle S, Takahashi H, Parsons P, Waters M (1999). Up-regulation of growth hormone receptor immunoreactivity in human melanoma. Anticancer Res.

[16] Wyatt D (1999). Melanocytic nevi in children treated with growth hormone. Pediatrics.

[17] Caldarola G, Battista C, Pellicano R (2010). Melanoma onset after estrogen, thyroid, and growth hormone replacement therapy. Clin Ther.

[18] Handler MZ, Ross AL, Shiman MI, Elgart GW, Grichnik JM (2012). Potential Role of Human Growth Hormone in Melanoma Growth Promotion. Arch Dermatol.

[19] Sustarsic EG, Junnila RK, Kopchick JJ (2013). Human metastatic melanoma cell lines express high levels of growth hormone receptor and respond to GH treatment. Biochem Biophys Res Commun.

[20] Iyengar B (2012). Hormone Expression in Melanomas Related to Tumor Angiogenesis. J Solid Tumors.

[21] Chatzistamou I, Volakaki AA, Schally AV, Kiaris H, Kittas C (2008). Expression of growth hormone-releasing hormone receptor splice variant 1 in primary human melanomas. Regul Pept.

[22] Szalontay L, Schally AV, Popovics P, Vidaurre I, Krishan A, Zarandi M, Cai RZ, Klukovits A, Block NL, Rick FG (2014). Novel GHRH antagonists suppress the growth of human malignant melanoma by restoring nuclear p27 function. Cell Cycle.

[23] Argetsinger LS, Campbell GS, Yang X, Witthuhn BA, Silvennoinen O, Ihle JN, Carter-Su C (1993). Identification of JAK2 as a growth hormone receptor-associated tyrosine kinase. Cell.

[24] VanderKuur JA, Wang X, Zhang L, Campbell GS, Allevato G, Billestrup N, Norstedt G, Carter-Su C (1994). Domains of the growth hormone receptor required for association and activation of JAK2 tyrosine kinase. J Biol Chem.

[25] Harding PA, Wang X, Okada S, Chen WY, Wan W, Kopchick JJ (1996). Growth hormone (GH) and a GH antagonist promote GH receptor dimerization and internalization. J Biol Chem.

[26] Carter-Su C, King AP, Argetsinger LS, Smit LS, Vanderkuur J, Campbell GS (1996). Signalling pathway of GH. Endocr J.

[27] A1 Sotiropoulos, Moutoussamy S, Renaudie F, Clauss M, Kayser C, Gouilleux F, Kelly PA FJ (1996). Differential activation of Stat3 and Stat5 by distinct regions of the growth hormone receptor. Mol Endocrinol.

[28] Campbell GS (1997). Growth-hormone signal transduction. J Pediatr.

[29] Liang L, Zhou T, Jiang J, Pierce JH, Gustafson TA, Frank SJ (1999). Insulin receptor substrate-1 enhances growth hormone-induced proliferation. Endocrinology.

[30] Piwien-Pilipuk G, Huo JS, Schwartz J (2002). Growth hormone signal transduction. J Pediatr Endocrinol Metab.

[31] Manabe N, Kubota Y, Kitanaka A, Ohnishi H, Taminato T, Tanaka T (2006). Src transduces signaling via growth hormone (GH)-activated GH receptor (GHR) tyrosine-phosphorylating GHR and STAT5 in human leukemia cells. Leuk Res.

[32] Barclay JL, Kerr LM, Arthur L, Rowland JE, Nelson CN, Ishikawa M, D’Aniello EM, White M, Noakes PG, Waters MJ (2010). In Vivo Targeting of the Growth Hormone Receptor (GHR) Box1 Sequence Demonstrates that the GHR Does Not Signal Exclusively through JAK2. Mol Endocrinol.

[33] Mimeault M, Batra SK (2010). New advances on critical implications of tumor- and metastasis-initiating cells in cancer progression, treatment resistance and disease recurrence. Histol Histopathol.

[34] Sedek M, van der Velden LM, Strous GJ (2014). Multimeric growth hormone receptor complexes serve as signaling platforms. J Biol Chem.

[35] Tsao H, Chin L, Garraway LA, Fisher DE (2012). Melanoma : from mutations to medicine. Genes Dev.

[36] Brychtova S, Bezdekova M, Hirnak J, Sedlakova E, Tichy M, Brycht T (2011). Stromal Microenvironment Alterations in Malignant Melanoma. Research on Melanoma - A Glimpse into Current Directions and Future Trends. InTech.

[37] Heerboth S, Housman G, Leary M, Longacre M, Byler S, Lapinska K, Willbanks A, Sarkar S (2015). EMT and tumor metastasis. Clin Transl Med.

[38] Mitra A, Mishra L, Li S (2015). EMT, CTCs and CSCs in tumor relapse and drug-resistance. Oncotarget.

[39] Liang SQ, Marti TM, Dorn P, Froment L, Hall SRR, Berezowska S, Kocher G, Schmid RA, Peng RW (2015). Blocking the epithelial-to-mesenchymal transition pathway abrogates resistance to anti-folate chemotherapy in lung cancer. Cell Death Dis.

[40] Fischer KR, Durrans A, Lee S, Sheng J, Li F, Wong STC, Choi H, El Rayes T, Ryu S, Troeger J, Schwabe RF, Vahdat LT, Altorki NK (2015). Epithelial-to-mesenchymal transition is not required for lung metastasis but contributes to chemoresistance. Nature.

[41] Friedl P, Wolf K (2003). Tumour-cell invasion and migration: diversity and escape mechanisms. Nat Rev Cancer.

[42] van Zijl F, Krupitza G, Mikulits W (2011). Initial steps of metastasis: Cell invasion and endothelial transmigration. Mutat Res Mutat Res.

[43] Shaw LM (2005). Tumor cell invasion assays. Methods Mol Biol.

[44] Vinci M, Box C, Eccles SA (2015). Three-Dimensional (3D) Tumor Spheroid Invasion Assay. J Vis Exp.

[45] Borowicz S, Van Scoyk M, Avasarala S, Karuppusamy Rathinam MK, Tauler J, Bikkavilli RK, Winn RA (2014). The Soft Agar Colony Formation Assay. J Vis Exp.

[46] Thomas SJ, Snowden JA, Zeidler MP, Danson SJ (2015). The role of JAK/STAT signalling in the pathogenesis, prognosis and treatment of solid tumours. Br J Cancer.

[47] Mirmohammadsadegh A, Hassan M, Bardenheuer W, Marini A, Gustrau A, Nambiar S, Tannapfel A, Bojar H, Ruzicka T, Hengge UR (2006). STAT5 Phosphorylation in Malignant Melanoma Is Important for Survival and Is Mediated Through SRC and JAK1 Kinases. J Invest Dermatol.

[48] Kortylewski M, Jove R, Yu H (2005). Targeting STAT3 affects melanoma on multiple fronts. Cancer and Metastasis Reviews.

[49] Cao HH, Chu JH, Kwan HY, Su T, Yu H, Cheng CY, Fu XQ, Guo H, Li T, Tse AK, Chou GX, Mo HB, Yu ZL (2016). Inhibition of the STAT3 signaling pathway contributes to apigenin-mediated anti-metastatic effect in melanoma. Sci Rep.

[50] Niu G, Bowman T, Huang M, Shivers S, Reintgen D, Daud A, Chang A, Kraker A, Jove R, Yu H (2002). Roles of activated Src and Stat3 signaling in melanoma tumor cell growth. Oncogene.

[51] Yu H, Pardoll D, Jove R (2009). STATs in cancer inflammation and immunity: a leading role for STAT3. Nat Rev Cancer.

[52] Sinnberg T, Lasithiotakis K, Niessner H, Schittek B, Flaherty KT, Kulms D, Maczey E, Campos M, Gogel J, Garbe C, Meier F (2009). Inhibition of PI3K-AKT-mTOR Signaling Sensitizes Melanoma Cells to Cisplatin and Temozolomide. J Invest Dermatol.

[53] Babchia N, Calipel A, Mouriaux F, Faussat AM, Mascarelli F (2010). The PI3K/Akt and mTOR/P70S6K Signaling Pathways in Human Uveal Melanoma Cells: Interaction with B-Raf/ERK. Investig Opthalmology Vis Sci.

[54] Yan L, Suyi L, Peng C, Lu C, Ming Q, Suyu J (2011). The effects of recombinant human growth hormone on promoting tumor growth depend on the expression of growth hormone receptor in vivo. JOE.

[55] Zhang W, Qian P, Zhang X, Zhang M, Wang H, Wu M, Kong X, Tan S, Ding K, Perry JK, Wu Z, Cao Y, Lobie PE (2015). Autocrine/Paracrine Human Growth Hormone-stimulated MicroRNA 96-182-183 Cluster Promotes Epithelial-Mesenchymal Transition and Invasion in Breast Cancer. J Biol Chem.

[56] Talati PG, Gu L, Ellsworth EM, Girondo MA, Trerotola M, Hoang DT, Leiby B, Dagvadorj A, McCue PA, Lallas CD, Trabulsi EJ, Gomella L, Aplin AE (2015). Jak2-Stat5a/b Signaling Induces Epithelial-to-Mesenchymal Transition and Stem-Like Cell Properties in Prostate Cancer. Am J Pathol.

[57] Wendt MK, Balanis N, Carlin CR, Schiemann WP (2014). STAT3 and epithelial-mesenchymal transitions in carcinomas. JAK-STAT.

[58] Buonato JM, Lazzara MJ (2014). ERK1/2 Blockade Prevents Epithelial-Mesenchymal Transition in Lung Cancer Cells and Promotes Their Sensitivity to EGFR Inhibition. Cancer Res.

[59] Xia P, Xu XY (2015). PI3K/Akt/mTOR signaling pathway in cancer stem cells: from basic research to clinical application. Am J Cancer Res.

[60] Nagathihalli NS, Merchant NB (2012). Src-mediated regulation of E-cadherin and EMT in pancreatic cancer. Front Biosci.

[61] Brown-Borg HM, Bartke A (2012). GH and IGF1: Roles in Energy Metabolism of Long-Living GH Mutant Mice. Journals Gerontol Ser A Biol Sci Med Sci.

[62] Wennbo H, Törnell J (2000). The role of prolactin and growth hormone in breast cancer. Oncogene.

[63] Harvey S, Martínez-Moreno CG, Luna M, Arámburo C (2015). Autocrine/paracrine roles of extrapituitary growth hormone and prolactin in health and disease: An overview. Gen Comp Endocrinol.

[64] Kucera R, Treskova I, Vrzalova J, Svobodova S, Topolcan O, Fuchsova R, Rousarova M, Treska V, Kydlicek T (2014). Evaluation of IGF1 serum levels in malignant melanoma and healthy subjects. Anticancer Res.

[65] Yoshida M, Selvan S, McCue PA, DeAngelis T, Baserga R, Fujii A, Rui H, Mastrangelo MJ, Sato T (2014). Expression of insulin-like growth factor-1 receptor in metastatic uveal melanoma and implications for potential autocrine and paracrine tumor cell growth. Pigment Cell Melanoma Res.

[66] Antoniadis AG, Petridou ET, Antonopoulos CN, Dessypris N, Panagopoulou P, Chamberland JP, Adami HO, Gogas H, Mantzoros CS (2011). Insulin resistance in relation to melanoma risk. Melanoma Res.

[67] Capoluongo E (2011). Insulin-Like Growth Factor System and Sporadic Malignant Melanoma. Am J Pathol.

[68] Molhoek KR, Shada AL, Smolkin M, Chowbina S, Papin J, Brautigan DL, Slingluff CL (2011). Comprehensive analysis of receptor tyrosine kinase activation in human melanomas reveals autocrine signaling through IGF-1R. Melanoma Res.

[69] Wang H, Shen SS, Wang H, Diwan AH, Zhang W, Fuller GN, Prieto VG (2003). Expression of insulin-like growth factor-binding protein 2 in melanocytic lesions. J Cutan Pathol.

[70] Das SK, Bhutia SK, Azab B, Kegelman TP, Peachy L, Santhekadur PK, Dasgupta S, Dash R, Dent P, Grant S, Emdad L, Pellecchia M, Sarkar D (2013). MDA-9/Syntenin and IGFBP-2 promote angiogenesis in human melanoma. Cancer Res.

[71] Renehan AG, Zwahlen M, Minder C, O’Dwyer ST, Shalet SM, Egger M (2004). Insulin-like growth factor (IGF)-I, IGF binding protein-3, and cancer risk: systematic review and meta-regression analysis. Lancet.

[72] Xi Y, Nakajima G, Hamil T, Fodstad O, Riker A, Ju J (2006). Association of insulin-like growth factor binding protein-3 expression with melanoma progression. Mol Cancer Ther.

[73] Slomiany MG, Rosenzweig SA (2004). IGF-1-Induced VEGF and IGFBP-3 Secretion Correlates with Increased HIF-1α Expression and Activity in Retinal Pigment Epithelial Cell Line D407. Investig Opthalmology Vis Sci.

[74] Ekberg S, Luther M, Nakamura T, Jansson JO (1992). Growth hormone promotes early initiation of hepatocyte growth factor gene expression in the liver of hypophysectomized rats after partial hepatectomy. J Endocrinol.

[75] Vergani E, Vallacchi V, Frigerio S, Deho P, Mondellini P, Perego P, Cassinelli G, Lanzi C, Testi MA, Rivoltini L, Bongarzone I, Rodolfo M (2011). Identification of MET and SRC Activation in Melanoma Cell Lines Showing Primary Resistance to PLX4032. Neoplasia.

[76] Wilson TR, Fridlyand J, Yan Y, Penuel E, Burton L, Chan E, Peng J, Lin E, Wang Y, Sosman J, Ribas A, Li J, Moffat J (2012). Widespread potential for growth-factor-driven resistance to anticancer kinase inhibitors. Nature.

[77] Tsao H, Chin L, Garraway LA, Fisher DE (2012). Melanoma: from mutations to medicine. Genes Dev.

[78] Girotti MR, Pedersen M, Sanchez-Laorden B, Viros A, Turajlic S, Niculescu-Duvaz D, Zambon A, Sinclair J, Hayes A, Gore M, Lorigan P, Springer C, Larkin J (2013). Inhibiting EGF Receptor or SRC Family Kinase Signaling Overcomes BRAF Inhibitor Resistance in Melanoma. Cancer Discov.

[79] Chattopadhyay C, Ellerhorst JA, Ekmekcioglu S, Greene VR, Davies MA, Grimm EA (2012). Association of activated c-Met with NRAS-mutated human melanomas. Int J Cancer.

[80] Cao HH, Cheng CY, Su T, Fu XQ, Guo H, Li T, Tse AK, Kwan HY, Yu H, Yu ZL (2015). Quercetin inhibits HGF/c-Met signaling and HGF-stimulated melanoma cell migration and invasion. Mol Cancer.

[81] Maverakis E, Cornelius LA, Bowen GM, Phan T, Patel FB, Fitzmaurice S, He Y, Burrall B, Duong C, Kloxin AM, Sultani H, Wilken R, Martinez SR (2015). Metastatic melanoma - a review of current and future treatment options. Acta Derm Venereol.

[82] Perry JK, Mohankumar KM, Emerald BS, Mertani HC, Lobie PE (2008). The Contribution of Growth Hormone to Mammary Neoplasia. J Mammary Gland Biol Neoplasia.

[83] Bougen NM, Steiner M, Pertziger M, Banerjee A, Brunet-Dunand SE, Zhu T, Lobie PE, Perry JK (2012). Autocrine human GH promotes radioresistance in mammary and endometrial carcinoma cells. Endocr Relat Cancer.

[84] Grimbly C, Martin B, Karpinski E, Harvey S (2009). Growth hormone production and action in N1E-115 neuroblastoma cells. J Mol Neurosci.

[85] Conway-Campbell BL, Wooh JW, Brooks AJ, Gordon D, Brown RJ, Lichanska AM, Chin HS, Barton CL, Boyle GM, Parsons PG, Jans DA, Waters MJ (2007). Nuclear targeting of the growth hormone receptor results in dysregulation of cell proliferation and tumorigenesis. Proc Natl Acad Sci U S A.

[86] Herington AC (2012). Signal Transduction Mechanisms Underlying Growth Hormone Receptor Action. Open Endocrinol J.

[87] Guevara-Aguirre J, Balasubramanian P, Guevara-Aguirre M, Wei M, Madia F, Cheng CW, Hwang D, Martin-Montalvo A, Saavedra J, Ingles S, de Cabo R, Cohen P, Longo VD (2011). Growth Hormone Receptor Deficiency Is Associated with a Major Reduction in Pro-Aging Signaling, Cancer, and Diabetes in Humans. Sci Transl Med.

[88] Laron Z (2004). Laron Syndrome (Primary Growth Hormone Resistance or Insensitivity): The Personal Experience 1958-2003. J Clin Endocrinol Metab.

[89] Melmed S (2001). Clinical perspective: Acromegaly and Cancer: Not a Problem?. J Clin Endocrinol Metab.

[90] Jenkins PJ, Besser M (2001). Clinical perspective: Acromegaly and Cancer: A Problem. J Clin Endocrinol Metab.

[91] Baris D, Gridley G, Ron E, Weiderpass E, Mellemkjaer L, Ekbom A, Olsen JH, Baron JA, Fraumeni JF (2002). Acromegaly and cancer risk: a cohort study in Sweden and Denmark. Cancer Causes Control.

[92] Renehan AG, Brennan BM (2008). Acromegaly, growth hormone and cancer risk. Best Pract Res Clin Endocrinol Metab.

[93] Baylin SB (2001). Aberrant patterns of DNA methylation, chromatin formation and gene expression in cancer. Hum Mol Genet.

[94] Sharma A, Kuzu OF, Nguyen FD, Sharma A, Noory M (2015). Current State of Animal (Mouse) Modeling in Melanoma Research. Cancer Growth Metastasis.

[95] Bentov I, Damodarasamy M, Plymate S, Reed MJ (2013). B16/F10 tumors in aged 3D collagen in vitro simulate tumor growth and gene expression in aged mice in vivo. Vitr Cell Dev Biol Anim.

[96] Chien CH, Lee MJ, Liou HC, Liou HH, Fu WM (2016). Growth hormone is increased in the lungs and enhances experimental lung metastasis of melanoma in DJ-1 KO mice. BMC Cancer.

[97] Goffin V, Touraine P, Pichard C, Bernichtein S, Kelly PA (1999). Should prolactin be reconsidered as a therapeutic target in human breast cancer?. Molecular and Cellular Endocrinology.

[98] Goffin V, Binart N, Touraine P, Kelly PA (2002). Prolactin: the new biology of an old hormone. Annu Rev Physiol.

[99] Jacobson EM (2011). Prolactin in breast and prostate cancer: molecular and genetic perspectives. Discov Med.

[100] Satyamoorthy K, Li G, Vaidya B, Patel D, Herlyn M (2001). Insulin-like growth factor-1 induces survival and growth of biologically early melanoma cells through both the mitogen-activated protein kinase and beta-catenin pathways. Cancer Res.

[101] Pollak M (2008). Insulin, insulin-like growth factors and neoplasia. Best Pract Res Clin Endocrinol Metab.

[102] Haisa M (2013). The type 1 insulin-like growth factor receptor signalling system and targeted tyrosine kinase inhibition in cancer. J Int Med Res.

[103] Subramani R, Lopez-Valdez R, Arumugam A, Nandy S, Boopalan T, Lakshmanaswamy R, Languino LR (2014). Targeting Insulin-Like Growth Factor 1 Receptor Inhibits Pancreatic Cancer Growth and Metastasis. PLoS One.

[104] Wise TL (2006). Delayed Onset of Igf2-Induced Mammary Tumors in Igf2r Transgenic Mice. Cancer Res.

[105] Lee JT, Brafford P, Herlyn M (2008). Unraveling the Mysteries of IGF-1 Signaling in Melanoma. JournalofInvestigativeDermatology.

[106] Hilmi C, Larribere L, Giuliano S, Bille K, Ortonne J-P, Ballotti R, Bertolotto C (2008). IGF1 promotes resistance to apoptosis in melanoma cells through an increased expression of BCL2, BCL-X(L), and survivin. J Invest Dermatol.

[107] King H, Aleksic T, Haluska P, Macaulay V (2014). Can we unlock the potential of IGF-1R in cancer therapy?. Cancer Treat Rev.

[108] Clemmons DR (2007). Modifying IGF1 activity: an approach to treat endocrine disorders, atherosclerosis and cancer. Nat Rev Drug Discov.

[109] Denduluri SK, Idowu O, Wang Z, Liao Z, Yan Z, Mohammed MK, Ye J, Wei Q, Wang J, Zhao L, Luu HH (2015). Insulin-like growth factor (IGF) signaling in tumorigenesis and the development of cancer drug resistance. Genes Dis.

[110] Lee C, Safdie FM, Raffaghello L, Wei M, Madia F, Parrella E, Hwang D, Cohen P, Bianchi G, Longo VD (2010). Reduced Levels of IGF-I Mediate Differential Protection of Normal and Cancer Cells in Response to Fasting and Improve Chemotherapeutic Index. Cancer Res.

[111] Kopchick JJ, List EO, Kelder B, Gosney ES, Berryman DE (2014). Evaluation of growth hormone (GH) action in mice: discovery of GH receptor antagonists and clinical indications. Mol Cell Endocrinol.

[112] Xue M, Cao X, Zhong Y, Kuang D, Liu X, Zhao Z, Li H (2012). Insulin-like growth factor-1 receptor (IGF-1R) kinase inhibitors in cancer therapy: advances and perspectives. Curr Pharm Des.

[113] Zhang K, Wong P, Salvaggio C, Salhi A, Osman I, Bedogni B (2016). Synchronized Targeting of Notch and ERBB Signaling Suppresses Melanoma Tumor Growth through Inhibition of Notch1 and ERBB3. J Invest Dermatol.

[114] Zerrad-Saadi A, Lambert-Blot M, Mitchell C, Bretes H, Collin de l’Hortet A, Baud V, Chereau F, Sotiropoulos A, Kopchick JJ, Liao L, Xu J, Gilgenkrantz H, Guidotti JE (2011). GH Receptor Plays a Major Role in Liver Regeneration through the Control of EGFR and ERK1/2 Activation. Endocrinology.

[115] Joo A, Aburatani H, Morii E, Iba H, Yoshimura A (2004). STAT3 and MITF cooperatively induce cellular transformation through upregulation of c-fos expression. Oncogene.

[116] Wang W, Edington HD, Rao UNM, Jukic DM, Wang H, Shipe-Spotloe JM, Kirkwood JM (2008). STAT3 as a Biomarker of Progression in Atypical Nevi of Patients with Melanoma: Dose-Response Effects of Systemic IFNα Therapy. J Invest Dermatol.

[117] Pensa S, Watson CJ, Poli V (2009). Stat3 and the Inflammation/Acute Phase Response in Involution and Breast Cancer. J Mammary Gland Biol Neoplasia.

[118] Ohanna M, Cheli Y, Bonet C, Bonazzi VF, Allegra M, Giuliano S, Bille K, Bahadoran P, Giacchero D, Philippe Lacour J, Boyle GM, Hayward NF, Bertolotto C (2013). Secretome from senescent melanoma engages the STAT3 pathway to favor reprogramming of naive melanoma towards a tumor-initiating cell phenotype. Oncotarget.

[119] Herrington J, Smit LS, Schwartz J, Carter-Su C (2000). The role of STAT proteins in growth hormone signaling. Oncogene.

[120] Jin H, Lanning NJ, Carter-Su C (2008). But Not Src Family Kinases, Is Required for STAT, ERK, and Akt Signaling in Response to Growth Hormone in Preadipocytes and Hepatoma Cells. Mol Endocrinol.

[121] Lesinski GB, Valentino D, Hade EM, Jones S, Magro C, Chaudhury AR, Walker MJ, Carson WE (2005). Expression of STAT1 and STAT2 in malignant melanoma does not correlate with response to interferon-alpha adjuvant therapy. Cancer Immunol Immunother.

[122] Wellbrock C, Weisser C, Hassel JC, Fischer P, Becker J, Vetter CS, Behrmann I, Kortylewski M, Heinrich PC, Schartl M (2005). STAT5 contributes to interferon resistance of melanoma cells. Curr Biol.

[123] Hassel JC, Winnemöller D, Schartl M, Wellbrock C (2008). STAT5 contributes to antiapoptosis in melanoma. Melanoma Res.

[124] Reddy EP, Korapati A, Chaturvedi P, Rane S (2000). IL-3 signaling and the role of Src kinases, JAKs and STATs: a covert liaison unveiled. Oncogene.

[125] Brandner JM, Haass NK (2013). Melanoma's connections to the tumour microenvironment. Pathology.

[126] Voulgari A, Pintzas A (2009). Epithelial-mesenchymal transition in cancer metastasis: Mechanisms, markers and strategies to overcome drug resistance in the clinic. Biochim Biophys Acta.

[127] Aksamitiene E, Hoek JB, Kholodenko B, Kiyatkin A (2007). Multistrip Western blotting to increase quantitative data output. Electrophoresis.

[128] Borra RC, Lotufo MA, Gagioti SM, Barros FDM, Andrade PM (2009). A simple method to measure cell viability in proliferation and cytotoxicity assays. Braz Oral Res.

[129] Kramer N, Walzl A, Unger C, Rosner M, Krupitza G, Hengstschläger M, In Dolznig H (2013). vitro cell migration and invasion assays. Mutat Res Mutat Res.

