# Supplementary material for: Targeting growth hormone receptor in human melanoma cells attenuates tumor progression and epithelial mesenchymal transition via suppression of multiple oncogenic pathways
Source: Oncotarget. 2017 Feb 16;8(13):21579–98. doi: 10.18632/oncotarget.15375 (PMC5400608; doi:10.18632/oncotarget.15375)
Supplement: Supplementary file 1 [file oncotarget-08-21579-s001.pdf]

Targeting growth hormone receptor in human melanoma cells attenuates tumor progression and epithelial mesenchymal transition via suppression of multiple oncogenic pathways

SUPPLEMENTARY FIGURES

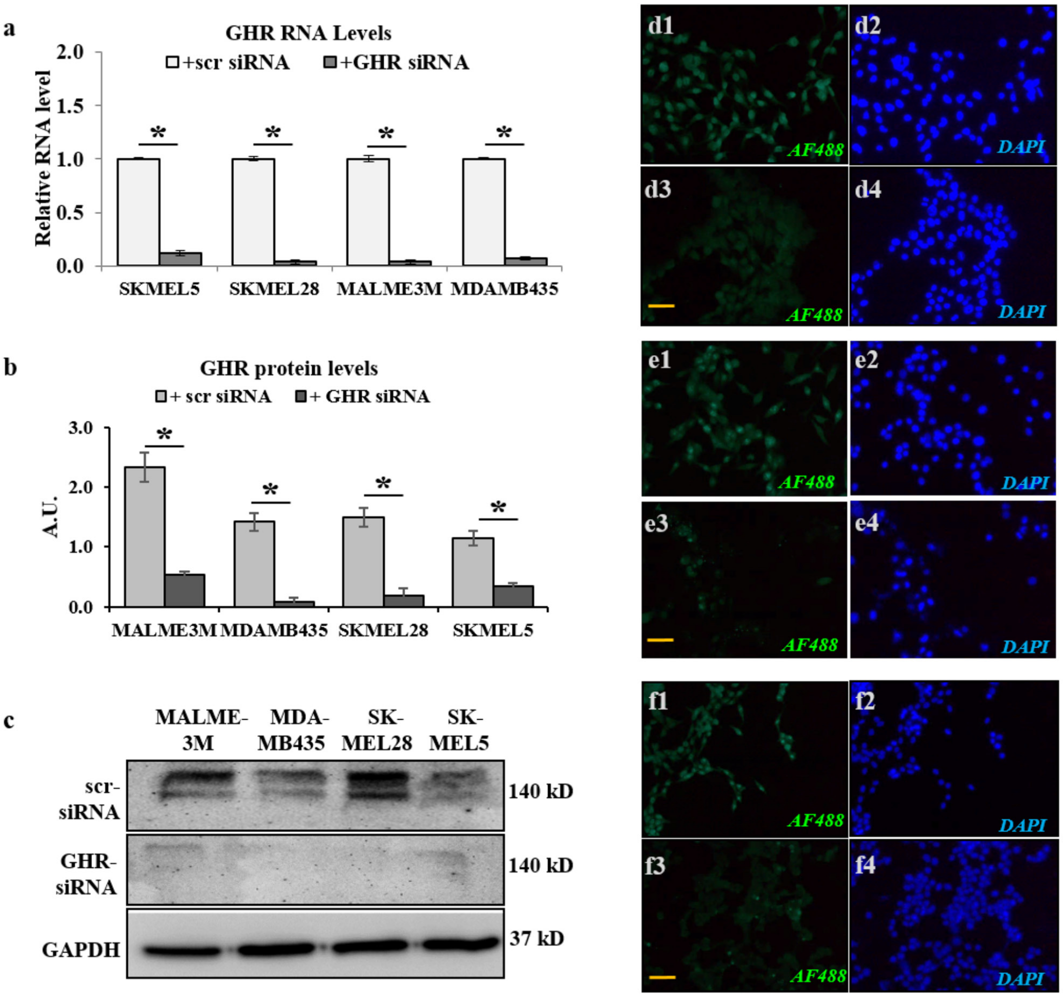

Supplementary Figure 1: si-RNA mediated knock-down of growth hormone receptor (GHR) expression in human melanoma cells. a. RT-qPCR analysis of GHR RNA levels in SKMEL-28, SKMEL-5, MDAMB-435 and MALME-3M melanoma cells transfected with scramble (scr)- or GHR-siRNA. RNA was collected 48 hr. post-transfection. Almost 90% reduction in mRNA levels was achieved in all four cell lines. Expressions were normalized against expression of actin and GAPDH as reference genes. [*, $p < 0.05$, Wilcoxon sign rank test, $n = 6$] **b.** Densitometry analyses of the protein level expression of GHR in scr- or GHR-siRNA transfected samples, from western-blot (WB) analyses of cell lysates collected 60 hr. post-transfection. Significant decrease in GHR expression was achieved by siRNA-mediated GHR knockdown. [*, $p < 0.05$, paired t-test, $n = 3$] **c.** Representative images of WB analyses of GHR in four melanoma cell lines. WB was performed using appropriate antibodies. Densitometry analyses of individual blots was performed using ImageJ software and the ratio of phosphorylated vs. total protein levels against untreated scr-siRNA transfected controls. **d-f.** GHR expression is abrogated following siRNA mediated GHRKD in melanoma cells. SKMEL-28 (d), MALME-3M (e), and MDAMB-435 (f) cells transfected with scr- or GHR specific siRNA. In each of the four boxes, *1* and *2* show melanoma cells transfected with scr-siRNA while the *3* and *4* show melanoma cells transfected with GHR-siRNA. In each box the left column shows GHR levels detected using AlexaFluor(AF)-488 (green) -conjugated (goat) secondary antibody to rabbit IgG specific for human GHR. while the right column shows the same cells' cellular DNA stained with DAPI (blue) From the average of four pictures per cell, maximum GHR-specific fluorescent signal was in the order of SKMEL-28 > MALME-3M > MDAMB-435 > SKMEL-5 (data not shown) – a trend also seen in WB analyses. ICC/IF was performed on cells 48 hours after transfection. Bar represents 500 um.

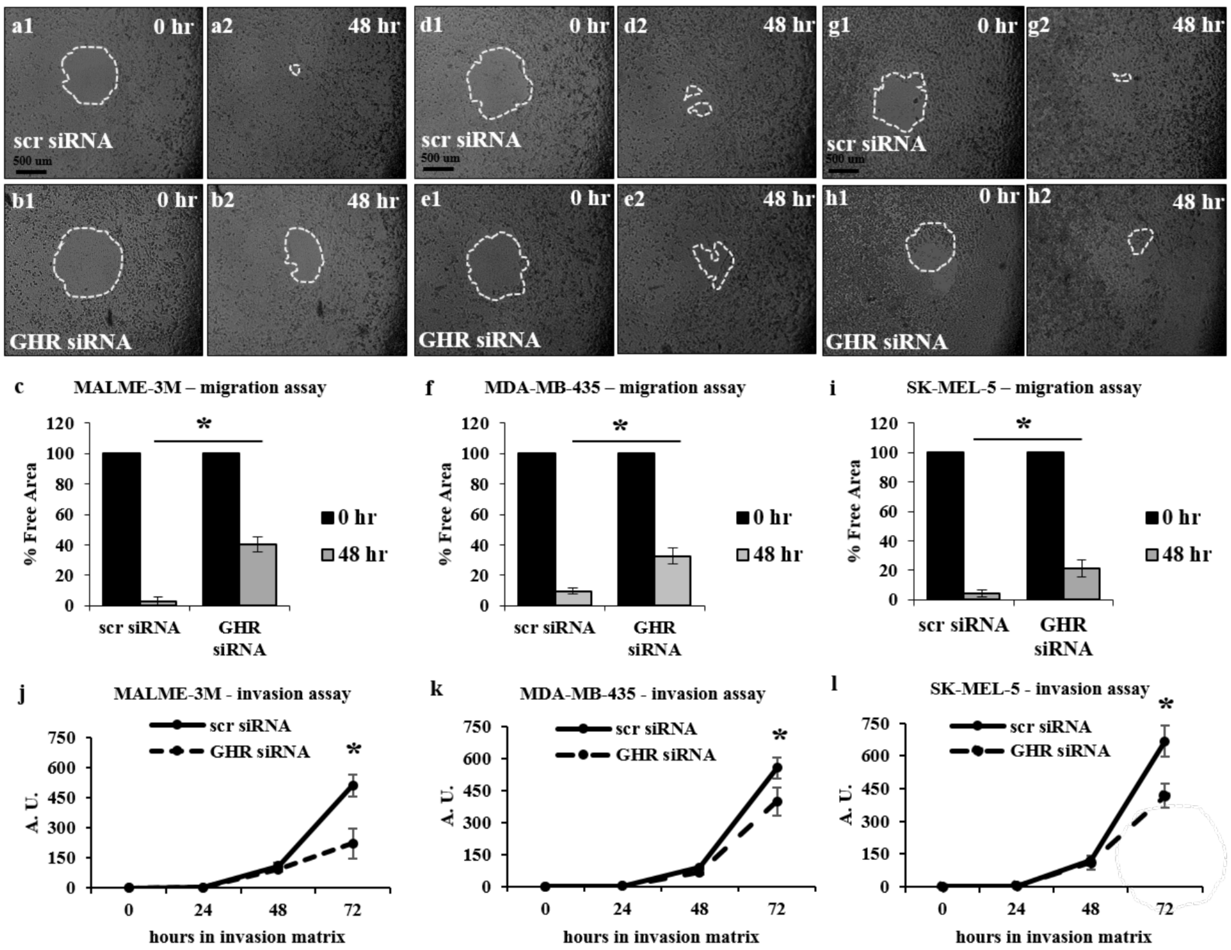

Supplementary Figure 2: GHR knock-down attenuates migration and invasion in human melanoma cells. MALME-3M **a-c.** MDA-MB-435 **d-f.** and SK-MEL-5 **g-i.** cells transfected with scr- or GHR-siRNA were allowed to migrate into a 0.68 mm circular spot at the center of the well, in presence of 50 ng/mL hGH for up to 48 hr. The percentage free area was calculated using ImageJ software and reflected the decrease/inhibition in migration. A significant decrease in migration was noted following GHR-KD. MALME-3M **(j)**, MDA-MB-435 **k.** and SK-MEL-5 **l.** cells transfected with scramble (scr)- or GHR-siRNA were seeded onto U-bottom 96-well plates at 5000 cells/well and allowed to form a spheroid. A hydrogel invasion matrix was added above the spheroid and cells were monitored for up to 72 hr. in presence of 50 ng/mL hGH. Total pixels representing structural extensions from the spheroid were calculated using ImageJ software and reflected the invasive ability of the melanoma cells. A significant decrease in spheroid invasion was noted following GHRKD. [*, $p < 0.05$, Students t-test, n = 3].

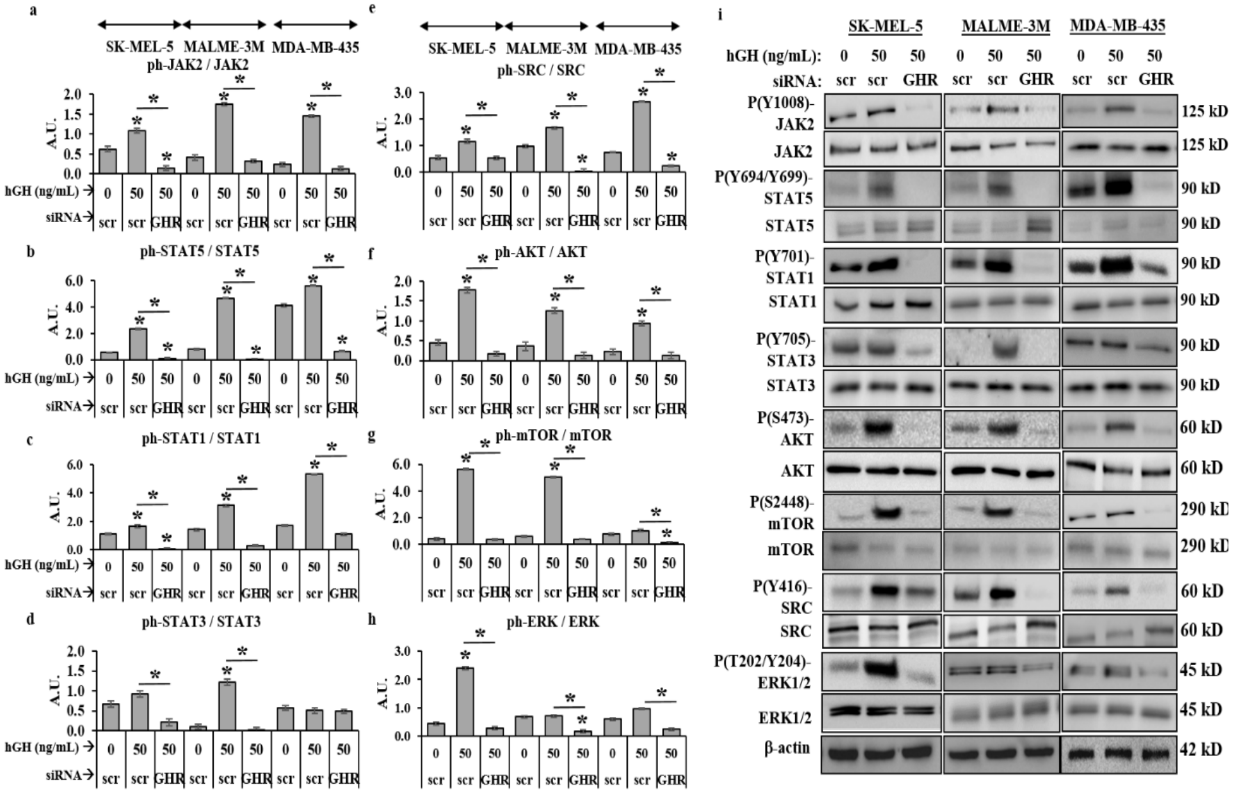

Supplementary Figure 3: GH-excess promotes and GHRKD attenuates phosphorylation levels of multiple critical intracellular signaling pathways in human melanoma cells. Comparison of western blot (WB) analyses of phosphorylation levels of **a.** JAK2, **b.** STAT5, **c.** STAT1, **d.** STAT3, **e.** SRC, **f.** AKT, **g.** mTOR and **h.** ERK1/2, in excess human-GH treated or GHRKD human melanoma cell lysates. MALME-3M, MDA-MB-435 and SK-MEL-5 cells, 24 hr post-transfection with either scramble (scr)-siRNA or GHR-siRNA were treated for ten mins with GH and lysed as described. **i.** Representative images of WB analyses of GHR in four melanoma cell lines. WB was performed using appropriate antibodies. Densitometry analyses of individual blots was performed using ImageJ software and the ratio of phosphorylated vs. total protein levels against untreated scr-siRNA transfected controls. Overall, excess GH increased while GHRKD decreased phosphorylation states. Blots from individual experiments were quantified and the mean of three blots per antibody was taken. Protein levels were normalized against expression of β-actin. [*, $p < 0.05$, Students t test, $n = 3$].

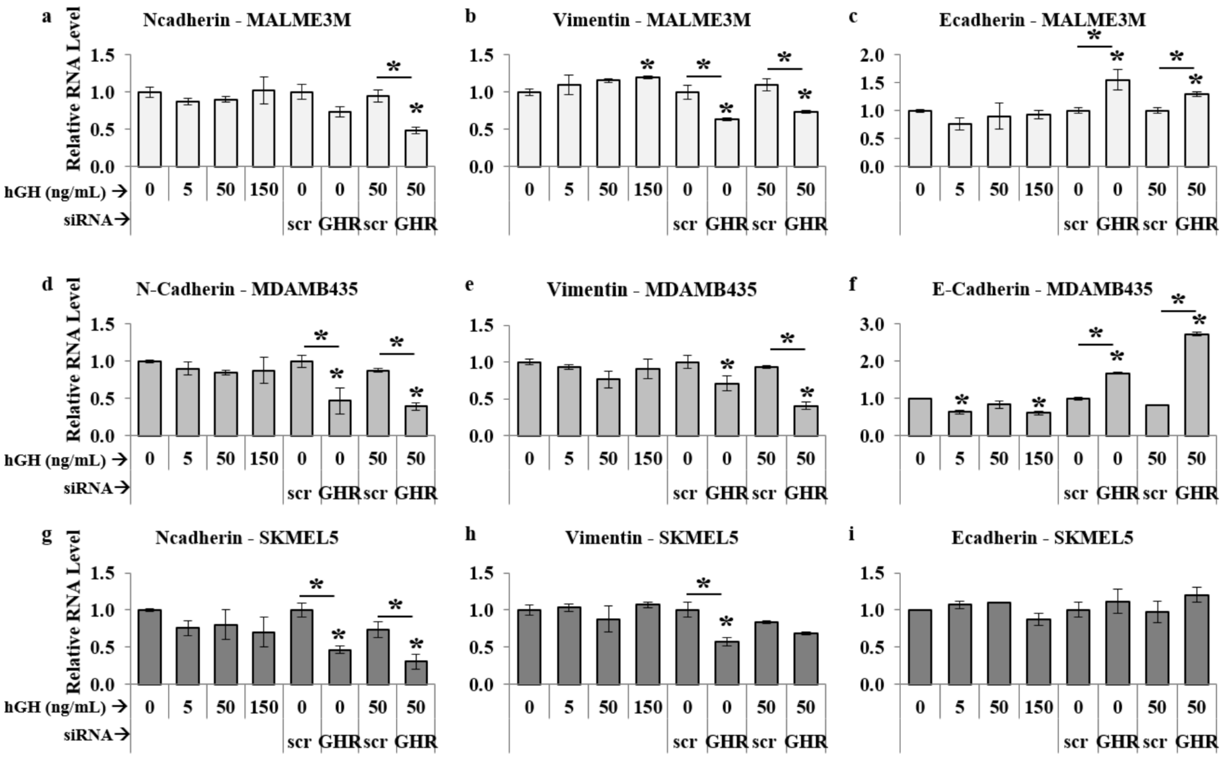

Supplementary Figure 4: GH-excess promotes and GHRKD attenuates RNA levels of markers of epithelial mesenchymal transition (EMT) in human melanoma cells. Relative RNA expression in MALME-3M **a-c.** MDA-MB-435 **d-f.** and SK-MEL-5 **g-i.** was quantified for N-cadherin (a, d, g), vimentin (b, e, h) and E-cadherin (c, f, i) following addition of 5, 50 and 150 ng/mL hGH or 24 hr following GHRKD, in presence or absence of 0 and 50 ng/mL hGH. Overall, excess GH promoted while GHRKD reversed RNA levels of EMT markers in human melanoma cells. In all cases, RNA expressions were normalized against β-actin and GAPDH values as reference genes and compared against untreated control. [*, $p < 0.05$, Wilcoxon sign rank test, n = 4].

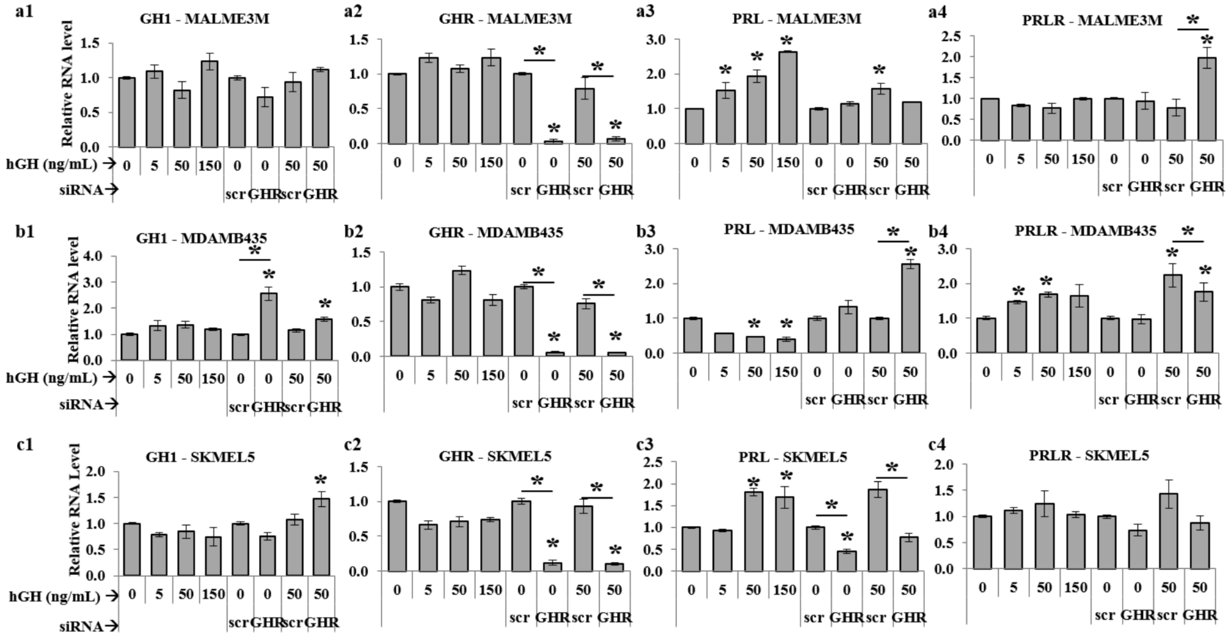

Supplementary Figure 5: RT-qPCR analysis of GH-GHR and PRL-PRLR pairs in human melanoma cells. Relative RNA levels of GH **a1, b1, c1.** GHR **a2, b2, c2.** PRL **a3, b3, c3.** and PRLR **a4, b4, c4.** following RT-qPCR of RNA extracted from MALME-3M (a), MDA-MB-435 (b) and SK-MEL-5 (c) cells following addition of 0, 5, 50 and 150 ng/mL hGH or following GHRKD, in presence or absence of 0 and 50 ng/mL hGH. Results are discussed in the text. In all cases, exogenous hGH treatment was for 24 hr. RNA levels were normalized against expression of β-actin and GAPDH as reference genes. [*, $p < 0.05$, Wilcoxon sign rank test, n = 4].

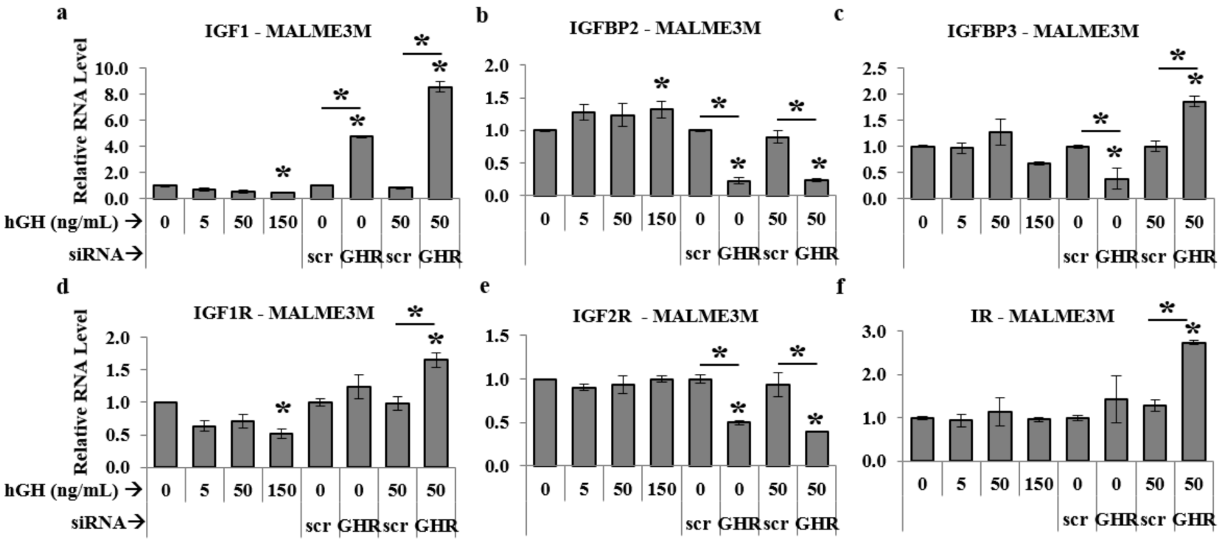

Supplementary Figure 6: RT-qPCR analysis of components of IGF axis in MALME-3M cells. Relative RNA levels of IGF1 **a.** IGFBP2 **b.** IGFBP3 **c.** IGF1R **d.** IGF2R **e.** and IR **f.** following RT-qPCR of RNA extracted from MALME-3M cells following addition of 0, 5, 50 and 150 ng/mL hGH or following GHRKD, in presence or absence of 0 and 50 ng/mL hGH. Results are discussed in the text. In all cases, exogenous hGH treatment was for 24 hr. RNA levels were normalized against expression of β-actin and GAPDH as reference genes. [*, $p < 0.05$, Wilcoxon sign rank test, n = 4].

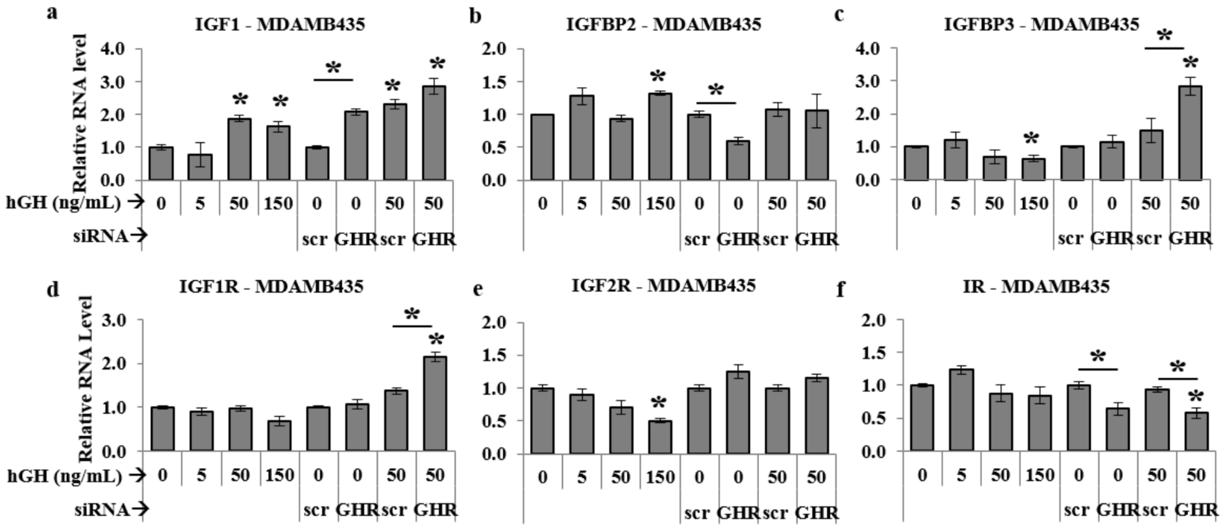

Supplementary Figure 7: RT-qPCR analysis of components of IGF axis in MDA-MB-435 cells. Relative RNA levels of IGF1 **a.** IGFBP2 **b.** IGFBP3 **c.** IGF1R **d.** IGF2R **e.** and IR **f.** following RT-qPCR of RNA extracted from MDA-MB-435 cells following addition of 0, 5, 50 and 150 ng/mL hGH or following GHRKD, in presence or absence of 0 and 50 ng/mL hGH. Results are discussed in the text. In all cases, exogenous hGH treatment was for 24 hr. RNA levels were normalized against expression of β-actin and GAPDH as reference genes. [*, $p < 0.05$, Wilcoxon sign rank test, n = 4].

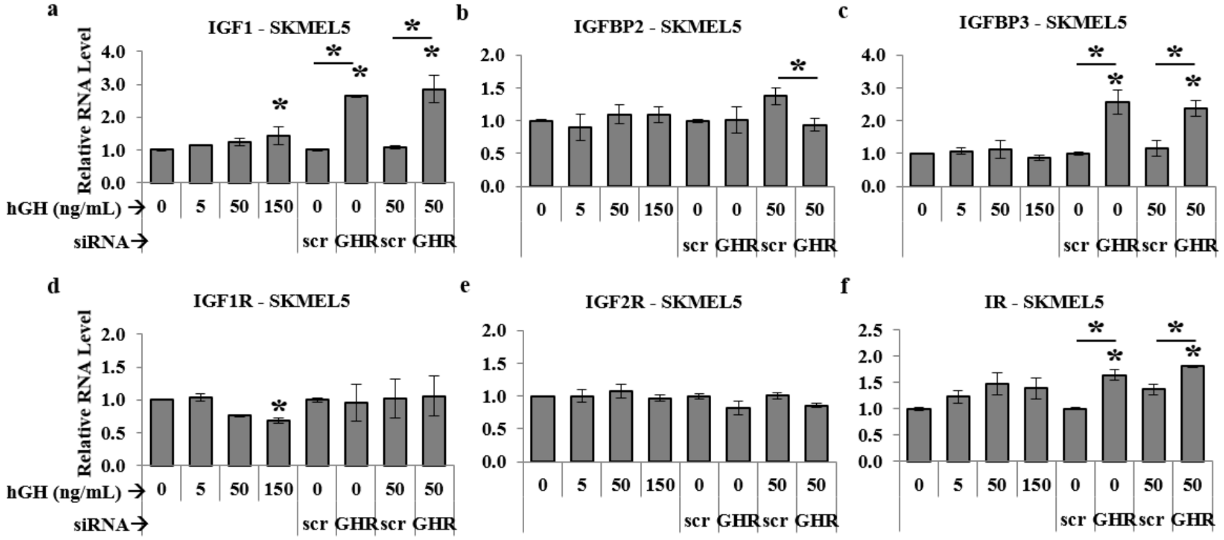

Supplementary Figure 8: RT-qPCR analysis of components of IGF axis in SK-MEL-5 cells. Relative RNA levels of IGF1 **a.** IGFBP2 **b.** IGFBP3 **c.** IGF1R **d.** IGF2R **e.** and IR **f.** following RT-qPCR of RNA extracted from SK-MEL-5 cells following addition of 0, 5, 50 and 150 ng/mL hGH or following GHRKD, in presence or absence of 0 and 50 ng/mL hGH. Results are discussed in the text. In all cases, exogenous hGH treatment was for 24 hr. RNA levels were normalized against expression of β-actin and GAPDH as reference genes. [*, $p < 0.05$, Wilcoxon sign rank test, n = 4].

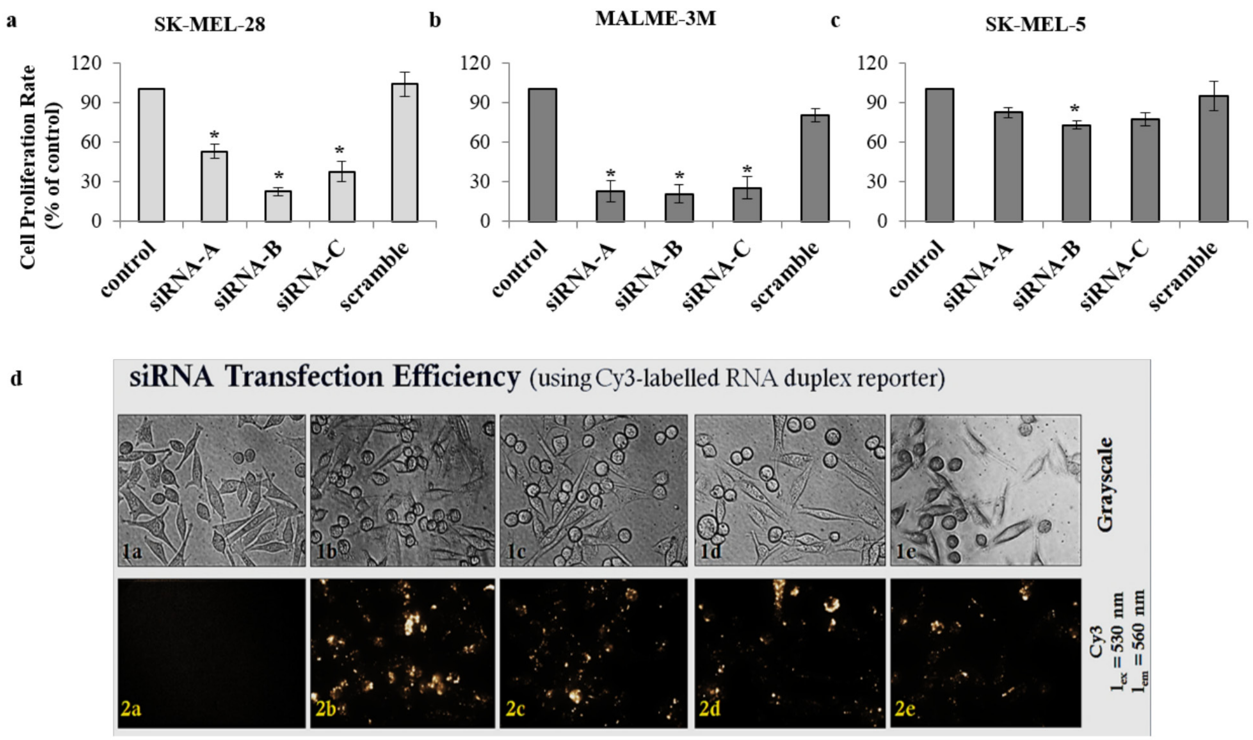

Supplementary Figure 9: Optimization of siRNA transfection of human melanoma cells. SK-MEL-28 **a.** MALME-3M **b.** and SK-MEL-5 **c.** cells were transfected with 20 nM of either GHR-siRNA or scramble siRNA for 24 hr. and cell proliferation was checked 60 hr. post-transfection using 0.04% resazurin (described in methods) and absorbance was read at 570 nm (600 nm = reference wavelength). Si-RNA-B was found to be optimum in bringing down GHR RNA levels most consistently across all cell lines. Similar data for MDA-MB-435 was observed (not shown here). [*, $p < 0.05$, Students t-test] **d.** SK-MEL-28 cells were plated at 10,000 cells/cm^2 and treated with either 20 nM GHR-specific siRNA (siRNA-A - 1b, 2b; siRNA-B – 1c,2c; siRNA-C, 1d, 2d) or scramble-siRNA (1e,2e). Untreated cells are treated with only transfection reagent (1a, 2a). Cells were photographed in grayscale (top row) and at 630 nm (bottom row). A Cy3-siRNA duplex at 10 nM was used as reporter. Red fluorescence indicates successful transfection. Identical results were obtained with SK-MEL-5, MALME-3M and MDA-MB-435 cells (data not shown here). Transfection was observed in more than 95% of cells.
